# Transport Number Determination and Relevance for Lithium Metal Batteries Using Localized Highly Concentrated Electrolytes

**DOI:** 10.1021/acs.chemmater.4c03067

**Published:** 2025-03-17

**Authors:** Hafiz Ahmad Ishfaq, Carolina Cruz Cardona, Elena Tchernychova, Patrik Johansson, Miran Gaberšček, Robert Dominko, Sara Drvarič Talian

**Affiliations:** † Department of Materials Chemistry, National Institute of Chemistry, Hajdrihova 19, Ljubljana 1000, Slovenia; ‡ Faculty of Chemistry and Chemical Technology, University of Ljubljana, Večna pot 113, Ljubljana 1000, Slovenia; § ALISTORE - European Research Institute, CNRS FR 3104, 15 Rue Baudelocque, Amiens Cedex 80039, France; ∥ Department of Physics, Chalmers University of Technology, Gothenburg 412 96, Sweden

## Abstract

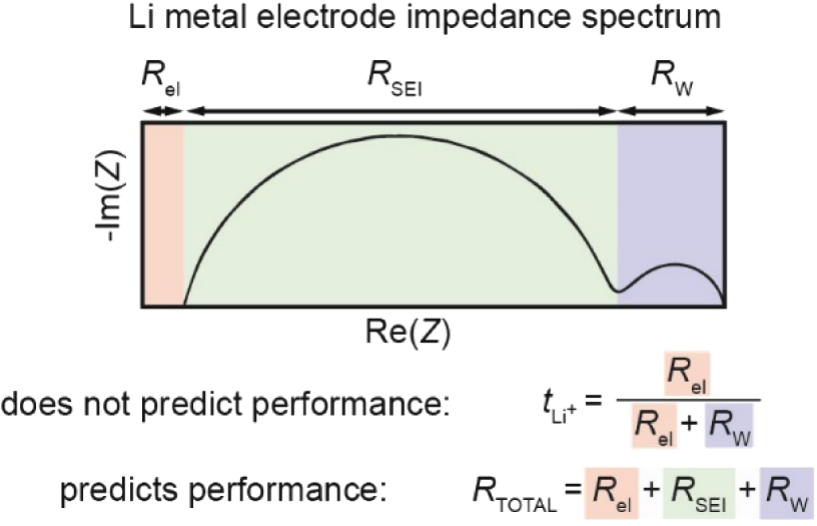

The lithium transport number  determination of fluorinated ether (1,2-(1,1,2,2-tetrafluoroethoxy) ethane, TFEE)-based localized highly concentrated electrolytes (LHCEs) with 1,2-dioxolane (DOL) and dimethoxyethane (DME) as solvents has been explored using molecular dynamics simulations, nuclear magnetic resonance spectroscopy, Bruce-Vincent’s method, and low-frequency electrochemical impedance spectroscopy (EIS). We showcase that the TFEE-DOL LHCE has a  as high as 0.65 but, on the other hand, exhibits low Coulombic efficiency (<90%) and poor stability *vs* Li metal anodes, *i.e.*, in a lithium metal battery (LMB) setting. In contrast, the TFEE-DME LHCE shows high Coulombic efficiency (98.9%) and stability, despite a much lower  (0.25). A significant migration resistance through the porous solid electrolyte interphase (SEI) for the former is the likely explanation, as revealed by EIS and assisted by scanning electron microscopy and X-ray photoelectron spectroscopy experiments. We thus find the interfacial properties at the Li metal anode to be more crucial than the ionic transport through the bulk of the electrolyte for LMB performance. We therefore propose that the focus should be put on the full (*operando*) impedance spectra of Li metal anodes in contact with electrolytes, since it enables the characterization of the interphase layer(s), rather than solely determining the (bulk)  of the electrolytes.

## Introduction

Lithium (Li) metal batteries (LMBs) have the potential to meet the increasing demand for high-performance batteries through the use of Li metal anodes, which offer high theoretical specific capacity (3860 mAh g^–1^) and low electrochemical potential (−3.04 V *vs* SHE).^1,2^ However, the thermodynamic instability of Li metal with the commonly used liquid electrolytes for lithium-ion batteries (LIBs) leads to the formation of an unstable solid electrolyte interphase (SEI). This instability refers to continuous formation of the SEI through the reaction between the electrolyte and electrode, as well as the consequences of the SEI’s weak mechanical properties, which results in the formation of high surface area Li (HSAL) over repeated cycles of cell operation and low Coulombic efficiency (CE) of LMBs.^3,4^ To overcome these challenges, highly concentrated electrolytes (HCEs) were developed and used,^5,6^ in which the concentration of “free” solvent is reduced and the cation first solvation shell is dominated by anions, in practice leading to stable anion-derived SEIs.^7^ The use of HCEs, however, is constrained by their high cost, due to the high concentration of salt, and poor electrode wettability, caused by their high viscosity, which also impairs ion transport. The addition of inert diluents (noncoordinating solvents), mainly fluorinated ethers, to HCEs, is a viable solution to address these issues.^8,9^ Although the addition of diluents decreases the electrolyte ionic conductivity because of their poor salt solubility, they offer high anodic stability, and, as nonsolvents they preserve the HCE local cation solvation structure at more moderate global electrolyte salt concentrations. This forms the so-called localized HCEs (LHCEs) – the electrolyte class applied herein.

A general problem for all liquid (nonaqueous) electrolytes is, however a lack of complete understanding of how battery performance is affected by the ion transport, which is mainly governed by three macroscopic properties: ionic conductivity,^10,11^ chemical diffusion coefficient,^12−14^ and Li^+^ transport number .^15−17^ Among these properties, , defined by the ratio of the ionic conductivity or diffusivity due to Li^+^*vs* that of other ion(s), has gained much attention by the battery research community.^18^ The research aims for a  as high as possible to minimize salt concentration gradients and thereby enable LMB fast-charging without ion transport limitations.^19−21^

The determination of  (by nonelectrochemical methods) is rather complex as it requires the strict assumption of an ideal electrolyte, *i.e. (i)* the salt is completely dissociated, *(ii)* all ions participate in migration and diffusion, and *(iii)* each ion moves independently and without the influence of other ions. To achieve ideality, the salt concentration in the electrolyte must be sufficiently low and the dielectric constant of the solvent sufficiently high. The upper threshold value for the ideality of aqueous electrolytes is 0.01 M for salts of monovalent ions.^22^ In reality, nonaqueous battery electrolytes have much higher salt concentrations (≥1 M). In particular, LHCEs can have a global salt concentration of 1 M, but locally they have much higher salt concentrations than conventional electrolytes, which renders ample formation of ion pairs and higher aggregates, and thus LHCEs deviate drastically from any ideal electrolyte.^8^

Deviations from ideality for battery electrolytes in general are well-founded. For instance, lithium hexafluorophosphate (LiPF_6_) /ethylene carbonate/dimethyl carbonate is a commonly used electrolyte for LIBs. The dissolution of LiPF_6_ in these carbonate solvents does not only produce Li^+^ and PF_6_^–^ ions but also ion pairs and higher aggregates ([*n*Li^+^–*m*PF_6_^–^]^*n*−^*^m^*).^18,23^ Goward et al. reported significant ion pairing for this electrolyte chemistry even at 0.2 M salt concentration.^24^ For any LHCE, we can rather safely assume a high degree of ion pairing, and likely also aggregate formation, but still some of the  values reported for LHCEs are remarkably high: 0.7 – 0.8,^18,25^ raising significant concerns with respect to the interpretation as they deviate from ideality.

The LHCEs are thus nonideal as a significant portion of the ions is expected to be nondissociated, and hence the determination of the transport number is challenging. In this regard, transference number  might be relevant.  is defined as the number of moles of Li^+^ (regardless of if the ion is completely dissociated or found in *e.g.*, ion pairs and/or aggregates) transferred by migration of a Coulomb of charge.^18,26^ In ideal electrolytes, the transport and transference numbers are identical.^18,27^ Many attempts have been made to measure the transference number in different LHCEs, and it has been found that the Li transference number decreases when diluents are added in HCEs.^16,28^ Although the authors have rigorously measured the transport properties of the LHCEs to describe the possible reasons for the transference number, they have rarely related the transport properties of the electrolyte to its electrochemical performance with Li metal.^16^

Importantly, the complexity of the solvation structures in LHCEs presents an increase in uncertainty as to whether the transference number is the best/correct parameter to describe the electrolyte transport. To elucidate this concept, consider a hypothetical LHCE containing fully dissociated salt species, Li^+^ and [A]^−^, alongside neutral [LiA] and charged complexes such as [Li_2_A]^+^ and [LiA_2_]^−^. When Li^+^-containing species reach the lithium metal electrode, they may participate in faradaic reactions. However, it remains uncertain whether all species contribute to such reactions and to what extent. For illustrative purposes, let us assume that, in this electrolyte, only Li^+^ and [LiA_2_]^−^ act as the active species responsible for charge transfer at the electrode. To define a transport-related parameter for this electrolyte—one that characterizes how the ionic current affects battery performance—we need to consider only the current contributions of active species Li^+^ and [LiA_2_]^−^. In this hypothetical LHCE system,  is defined based solely on the charge transferred by Li^+^, whereas the  considers the contributions from Li^+^, [Li_2_A]^+^, and [LiA_2_]^−^. Notably, neither of these definitions thus aligns with the actual conditions during battery operation, where only Li^+^ and [LiA_2_]^−^ are pertinent.

In this regard, the correct “transport number” would consider the percentage of charge carried by all species which contain Li and undergo the faradaic reaction on the electrode surface (defined as active species), which we call the *transport number of active ions*, *t*_active_. Note that this is not necessarily the same as the , since it is possible that a species contains Li and yet does not allow for faradaic reaction to take place on the electrode.

To address this, in this study, we have focused on LHCEs based on fluorinated ether 1,2-(1,1,2,2-tetrafluoroethoxy)ethane (TFEE) as diluent and 1,2-dimethoxyethane (DME) or 1,3-dioxolane (DOL) as solvents, which have shown promise as electrolytes for LMBs.^29,30^ We opted for the TFEE diluent because it is relatively inexpensive in comparison to other fluorinated ethers and is commercially available. The nonfluorinated ethers DME and DOL were chosen as cosolvents with TFEE (to dissolve Li salt) because they are commonly used solvents in LIB electrolytes.^31^ We determined  using various techniques: pulsed-field gradient (PFG) nuclear magnetic resonance (NMR) spectroscopy, molecular dynamics (MD) simulations, the Bruce-Vincent (BV) method, and electrochemical impedance spectroscopy (EIS). The advantages and disadvantages of each technique are elaborated upon, and finally we connect the observations made to several LMB performance measures, in order to guide future electrolyte development efforts.

## Experimental Section

### Materials

DME and DOL (HPLC grade, 99.9%) were purchased from Sigma-Aldrich, while TFEE (99%) was purchased from Apollo Scientific. Battery-grade lithium bis(trifluoromethanesulfonyl)imide (LiTFSI) was acquired from Sigma-Aldrich (99%). The carbon-coated lithium titanate spinel Li_4_Ti_5_O_12_ (LTO) powder was purchased from NEI Corporation. The carbon black (SUPER C65) was used as a conductive agent in the cathode slurries. The Li metal foil was from FMC (110 μm thick). The Cu foil (20 μm thick) was purchased from Goodfellow. Celgard 2320 was used as a separator in all cells. In some cases, glassy fiber separators (GF/A, Whatman) were also used as indicated.

### Electrolytes and Electrode Preparation and Cell Assembly

Solvent drying and electrolyte preparation were done under protective argon atmosphere in an MBraun glovebox, where O_2_ and H_2_O contents were followed and kept <1 ppm. To prepare the electrolytes, the DME and DOL solvents were dried with 4 Å molecular sieves for 5 days, refluxed overnight with Na/K alloy, and then purified by fractional distillation. The TFEE solvent was dried with 4 Å molecular sieves for 10 days. The water content in all solvents was determined by Karl Fischer titration (Mettler Toledo, C20) and was <0.6 ppm. For the preparation of electrolytes, both DME and DOL were first mixed in a 1:1 (v:v) ratio with TFEE. The LiTFSI salt was stoichiometrically weighed into a volumetric flask and dissolved in a small amount of prepared solvent mixture before the volumetric flask was filled to the marked line. The prepared LHCEs were 1 M LiTFSI in TFEE:DME (1:1, v:v) and 1 M LiTFSI in TFEE:DOL (1:1, v:v). For simplicity, we henceforth omit the volume ratios, and the two electrolytes are designated as 1 M LiTFSI in TFEE-DME and 1 M LiTFSI in TFEE-DOL, respectively.

The LTO cathode slurry was prepared by mixing 80 wt % active material, 10 wt % C65, and 10 wt % PVdF binder in NMP using ball milling (30 min, 300 rpm). The resulting slurry was cast onto Cu foil using a doctor blade applicator and dried under vacuum at 80 °C for 2 h. The LTO mass loading was *ca*. 7.4–7.6 mg cm^–2^ and the thickness was *ca*. 150 μm. The LTO cathodes were punched into discs with a diameter of 12 mm, pressed by a hydraulic press using a weight of 1.0 t for 30 s, and dried again under vacuum at 90 °C overnight before they were transferred to the glovebox.

All cell preparation was conducted inside an argon-filled MBraun glovebox. For the determination of the Coulombic efficiency (CE) of lithium stripping and plating, CR2032 coin cells were assembled using a Hohsen Corporation manual crimping tool. For all other cell assemblies, pouch cell casings with Cu contacts were employed. In most cell assemblies, the Li metal anodes were 14 mm in diameter and one Celgard 2320 separator was used, but in some cases, the separators were multiplied as indicated. The amount of electrolyte added was 10 μL per separator plus 10 μL for the Li anodes. In the Li||LTO cells, an additional 10 μL was added to account for the porosity of the electrode. Both of these LHCEs were tested under identical conditions (*i.e.*, same electrode mass loading, thickness, and electrolyte amount) to minimize the effect of testing conditions.

### Experimental Details for  Determination by Various Methods

#### NMR

^7^Li and ^19^F nuclear magnetic resonance spectroscopy (NMR) spectra were recorded using an Avance Neo 600 MHz spectrometer (Bruker). Coaxial 5 mm NMR tubes were used. The ^7^Li NMR spectra were measured using 1 M LiCl in D_2_O as an internal standard in an NMR coaxial tube. The internal standard for recording ^19^F NMR spectra was DMSO-*d*_*6*_ containing 0.03 wt % deuterated trifluoroacetic acid (CF_3_COOH) as solvent. Pulsed-field gradient (PFG) NMR was used to measure the Li^+^ and TFSI diffusion coefficients of the electrolytes. The PFG echo intensity was measured after executing a PFG NMR base pulse sequence and self-diffusivities were obtained by fitting the linearized version of the Stejskal–Tanner equation^32^ (Figure S1).
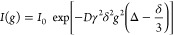
1

Where *I*(*g*) and *I*_0_ are the intensity of the PFG-echo profile at the gradient strength of *s* and 0 respectively, *D* is the diffusion coefficient of the ion, γ the gyromagnetic ratio, δ is the gradient duration, *g* is the gradient strength, and Δ is the gradient pulse interval (*i.e.*, the diffusion time). For ^7^Li and ^19^F NMR, the values for γ, δ, *g*, and Δ are listed in Table S1.  was calculated by the diffusivities obtained from PFG NMR through eq 2:
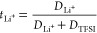
2

#### MD

Classical molecular dynamics (MD) simulations of the bulk electrolyte were performed to analyze the local structure and the mobilities of the species. This analysis involved calculating radial distribution functions (RDFs), coordination numbers (CNs), and self-diffusion coefficients. The simulations were conducted using the LAMMPS software^33^ with the nonpolarizable OPLS-AA force field (FF).^34^ Cubic simulation boxes, each containing 1500 molecules, were used for each electrolyte, with the details of the exact compositions in Table S2. Molecular topology files, along with Lennard-Jones and bonded parameters, were generated using the LigParGen server^35−37^ and the *fftool* package.^38^ To account for electronic screening and improve predictions of interionic interactions, a scaling factor of 0.8 was applied to the partial charges of ions due to the nonpolarizable nature of the FF.^39,40^ The simulation began with energy minimization using conjugate gradients, followed by a three-step equilibration process. First, an annealing approach was implemented for 2.5 ns, during which the system was heated to 600 K in increments of 30 K to overcome energy barriers and explore a wide conformational space. After maintaining the system at this elevated temperature to ensure thorough sampling, the temperature was slowly decreased to 300 K in steps of 30 K. This gradual cooling helped the system settle into a lower energy state, avoiding local minima and achieving a more stable configuration. The system was then equilibrated for 3 ns in the isothermal–isobaric ensemble (NPT), followed by an additional 8 ns in the canonical ensemble (NVT). Subsequently, an NVT production run was carried out for 10 ns. All simulations were performed at a temperature of 300 K and a pressure of 1 atm. A Nosé-Hoover thermostat was used with temperature and pressure damping parameters set to 100 and 1000 fs, respectively. Electrostatic interactions were calculated using the particle–particle-particle-mesh scheme, and periodic boundary conditions were applied in all directions. RDFs, CNs, and mean-square displacements (MSDs) for subsequent structural analysis were obtained from LAMMPS subroutines. The dynamic analysis is based on the calculation of self-diffusion coefficients (*D*_*i*_) from the MSDs using the Einstein relation^41^ as follows:
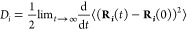
3where **R**_***i***_(t) is the position of the *i*-th at the time *t* and the brackets denote the ensemble average. The primary condition is that reasonable averages require long simulation times, as eq 3 assumes that individual ion movements are uncorrelated. Using eq 2,  can then be determined using  and *D*_TFSI_.

It is important to mention here that the self-diffusion coefficients calculated from MD simulations provide a measure of ion mobilities at the molecular level, capturing local interactions, such as solvation and ion pairing, as represented by the FF. However, they do not explicitly account for the macroscopic effects of concentration gradients or collective ion dynamics, which are related to chemical diffusion coefficients. The relationship between self-diffusion and chemical diffusion can be approximated using the Nernst–Einstein relation under ideal conditions, where ion–ion correlations are negligible. However, in the LHCEs studied here, deviations from ideality arise due to significant ion pairing and aggregate formation, which reduce the effective number of mobile species and alter the transport dynamics. This further highlights the importance of interpreting self-diffusion coefficients as foundational but incomplete descriptors of ion mobility in nonideal systems.^41^

#### Electrochemical Methods

 of the electrolytes was electrochemically measured using the Bruce-Vincent (BV) method.^42,43^ For this experiment, Li||Li symmetrical cells were allowed to stabilize for 24 h. The EIS spectra were then measured in the frequency range of 1 MHz to 1 Hz with a potential amplitude of 5 mV. The initial interfacial resistance (*R*_o_) was calculated from the EIS spectra. After this, the cells were subjected to a small polarization potential (Δ*V*) of 10 mV, from which initial (*I*_o_) and steady-state (*I*_s_) current were evaluated. After polarization, the EIS spectra were rerecorded to calculate the steady-state interfacial resistance (*R*_s_).  was calculated using eq 4.
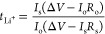
4

To obtain the  of the electrolytes, low-frequency EIS was also used.^44−46^ For this purpose, Li||Li cells with different numbers of Celgard separators (1×, 3×, and 9×) were assembled. The cells were then stabilized by measuring EIS in the frequency range of 1 MHz–1 mHz with the potential amplitude of 5 mV. After 24 h of stabilization, low-frequency measurements were performed in the range of 1 MHz to 0.1 mHz with a potential amplitude of 5 mV in a thermostat at 24 °C. The  was then determined using eq 5.
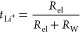
5

Where *R*_el_ is the bulk electrolyte resistance calculated from the real part of impedance at high frequencies and *R*_W_ is the real part of the diffusion impedance occurring at low frequencies.

#### Materials and Electrochemical Characterization

For *post-mortem* analysis, Li||Li cells were assembled with different electrolytes and EIS was measured at OCV for 1 or 100 h. After cell disassembly, the Li electrodes were carefully washed with a few drops of anhydrous DME (for the 1 M LiTFSI in TFEE-DME case) and DOL (for the 1 M LiTFSI in TFEE-DOL case) for about 20 s to remove the electrolyte salt and minimize unwanted damages to electrode surface due to handling. The morphology of the Li deposits was examined with scanning electron microscopy (SEM; SUPRA 35VP, Zeiss, Germany). The samples were transferred to the SEM with a vacuum transfer tool to ensure a protective atmosphere. The acceleration voltage was 1.5 kV.

X-ray photoelectron spectroscopy (XPS) was performed using a VersaProbe III AD (Phi, Chanhassen, US) equipped with a monochromatic Al-Kα1 X-ray source (1486.7 eV). Spectra were collected from a 200 μm in diameter spot size with the charge neutralizer activated to prevent differential charging, as the electrodes were mounted on a nonconductive double tape. High-resolution spectra were acquired with a pass energy of 69 eV and a step size of 0.05 eV. Charge neutralization was employed, and the energy scale of the XPS spectra was calibrated by setting the C 1*s* peak of carbon to a binding energy of 284.8 eV. Sputter-depth profiles of the SEI layer were obtained using alternating cycles of XPS analysis and sputtering with a focused 1 keV Ar beam. During each measurement cycle, the C 1*s* and F 1*s* regions were recorded with a step size of 0.13 eV and a pass energy of 69 eV. The spectra were analyzed using UlVAC-PHI Multipak software, with a binding energy error limit of ±0.2 eV for all peak fits. Shirley’s background correction was applied to all spectra. Instrumental broadening was assumed to be identical for all regions; therefore, the Gaussian fraction of each main peak was fixed at 90%. The full width at half-maximum (fwhm) for the fitted main peaks in the C 1*s* regions was set to 1.5 ± 20%. The determined fwhm and binding energy (BE) values for each resolved peak component, which dominated at specific sputter depths, were fixed for the batch-fitting procedure of the entire spectral series across all sputter depths.

The local solvation structures of the electrolytes were investigated using both Raman and NMR spectroscopy. The details of obtaining NMR spectra are described in the section above. The Ram II Raman spectrometer with an excitation laser of 785 nm and a resolution of 2 cm^–1^ was used to obtain Raman spectra of solvents and electrolytes by averaging over 200 samplings in the range 200–4000 cm^–1^.

The efficiency of Li metal plating and stripping was determined using Li||Cu cells with a current density of 0.5 mA cm^–2^ to a cutoff areal capacity of 0.5 mAh cm^–2^. The average Coulombic efficiencies (CEs) were additionally determined by applying the standard Aurbach method^47^ to the Li||Cu cells using the exact protocol described in the literature.^48^ The Li||LTO cells were tested in pouch cells with Cu contacts. These cells were tested in a voltage range of 1.0–2.5 V *vs* Li^+^/Li° at 0.6 C charge and discharge rates. We used Li||LTO cells because LTO is a zero-strain material that operates at a relatively low operating voltage^49^ – therefore not challenging the electrolyte’s oxidative stability. *Operando* EIS was carried out on Li||Li cells cycled at the same current density as used for the evaluation of CEs in Li||Cu cells, *i.e.*, 0.5 mA cm^–2^. Galvanostatic EIS was used with a current amplitude of 77 μA (*I*_DC_/10) in the frequency range of 1 MHz to 20 mHz with 14 spectra measured during one-half cycle, each spectrum measurement lasting for approximately 4.5 min. After 30 cycles of stripping and plating with *operando* EIS measurements, 30 min of OCV and PEIS measurements, with 5 mV (rms) potential amplitude, in the same frequency range was performed.

## Results and Discussion

**Figure 1:**
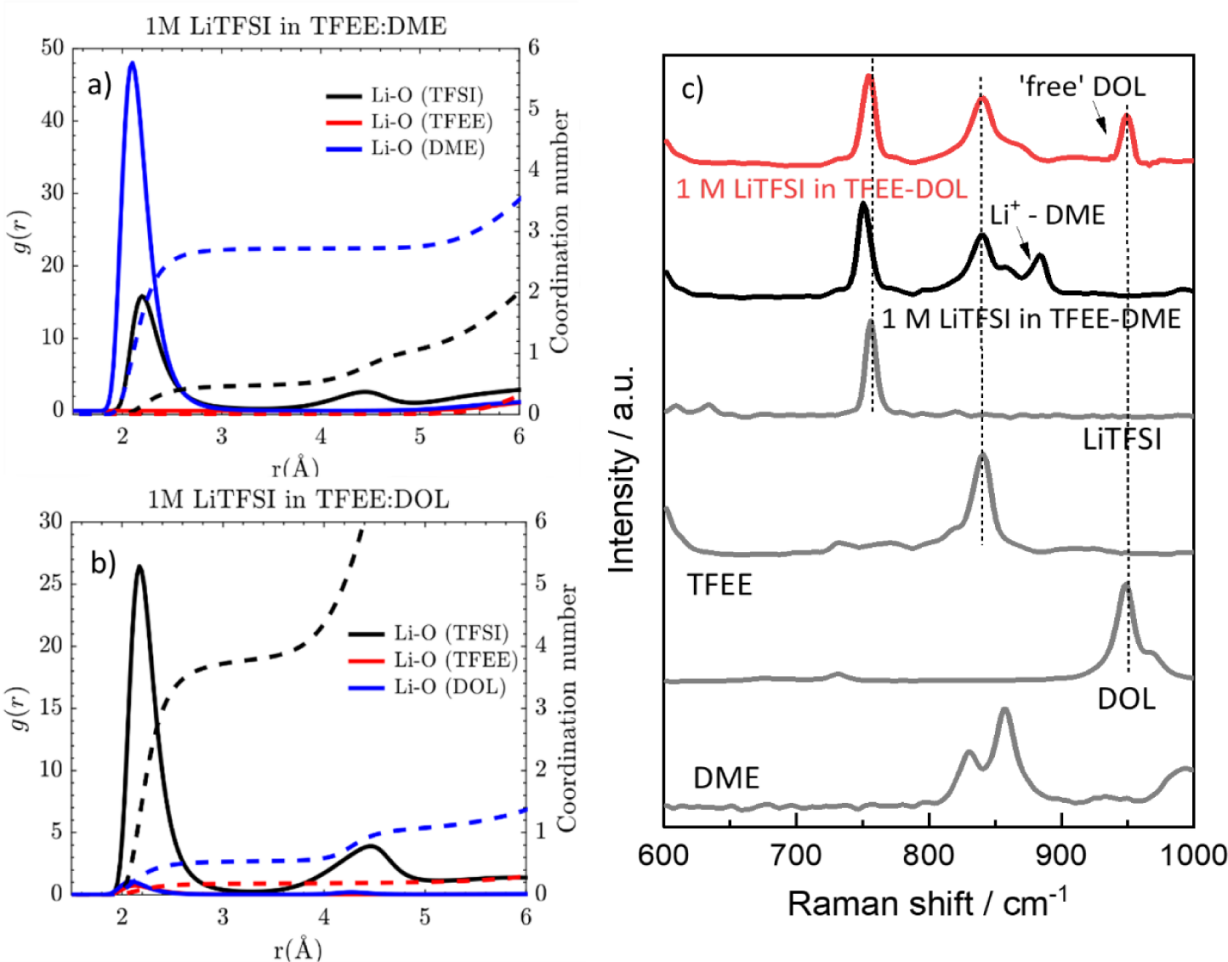
Li^+^-O radial distribution functions from the MD simulation trajectories for (a) 1 M LiTFSI in TFEE-DME and (b) 1 M in LiTFSI in TFEE-DOL. (c) Raman spectra of solvents, salt, and electrolytes.

First, we studied the local electrolyte solvation structures using MD simulations, Raman spectroscopy, and NMR spectroscopy. The calculated RDFs and CNs for Li^+^-O, divided up in Li^+^-O (TFSI), Li^+^-O (TFEE), Li^+^-O (DOL), and Li^+^-O (DME) (Table S3) show that in the 1 M LiTFSI in TFEE-DME electrolyte (Figure 1a), Li^+^ is mainly coordinated by DME oxygen atoms with a CN = 2.7 and weakly coordinated by the TFSI oxygen atoms with a CN = 0.48. In contrast, in the 1 M LiTFSI in TFEE-DOL electrolyte (Figure 1b), Li^+^ is predominantly coordinated by TFSI, with a CN = 3.8 by TFSI oxygens atoms. In addition, there are weak interactions between Li^+^-O (TFEE) and Li^+^-O (DOL) within the first cation solvation shell, with CNs of 0.18 and 0.54, respectively, indicating that DOL in particular interacts weakly with Li^+^ in the 1 M LiTFSI in TFEE-DOL electrolyte, in accordance with the literature.^31^ The local solvation structures of these electrolytes based on the MD simulations are schematically shown in Figure S2 and verified by Raman spectroscopy as shown in Figure 1c. According to this, both DME (peak at *ca*. 883 cm^–1^) and TFSI (*ca*. 748 cm^–1^) coordinate with Li^+^, indicating the formation of ion pairs as well as solvation by DME. In contrast, no strong solvation by DOL is observed for the 1 M LiTFSI in TFEE-DOL electrolyte, but the peaks of TFSI (*ca*. 756 cm^–1^), including ion pairs, and mainly unperturbed “free” DOL (*ca*. 949 cm^–1^) are present, largely confirming the MD results. The local electrolyte structures were also confirmed by NMR spectroscopy. An upfield shift in the ^7^Li NMR is observed when LiTFSI is dissolved in TFEE-DME *vs* the TFEE-DOL mixture, which could be due to stronger ion solvation or an increase in ion pairing.^50^^19^F NMR was measured to distinguish between these two phenomena and a downfield shift was observed going from TFEE-DME to TFEE-DOL electrolyte (Figure S3a,b). This signifies ion solvation of Li^+^ in 1 M LiTFSI in TFEE-DME electrolyte with DME solvent as well as TFSI anions and the strong interaction of Li^+^ with TFSI in the 1 M LiTFSI in TFEE-DOL electrolyte, which is consistent with both the MD simulations and the Raman spectroscopy results. In essence, Li^+^ is present in the form of ion pairs or higher aggregates in the electrolytes.

The MD simulations of the 1 M LiTFSI in TFEE-DME and 1 M LiTFSI in TFEE-DOL electrolytes show the latter to have higher Li^+^ mobility by the MSDs at longer time scales (Figure S4), which then is reflected in higher Li^+^ diffusion coefficients (Table S4). Using these diffusion coefficients to determine , it is slightly higher for the 1 M LiTFSI in TFEE-DOL electrolyte as compared to the 1 M LiTFSI in TFEE-DME electrolyte (0.50 *vs* 0.45). The self-diffusion of Li^+^ and TFSI using PFG-NMR (Table S4) again shows the 1 M LiTFSI in TFEE-DOL to have the higher Li^+^ and TFSI diffusion coefficients. Furthermore, the calculated transport numbers are quite similar: 0.51 and 0.48, respectively. However, the diffusion coefficients differ significantly (Tables S4 vs S5), which could be due to the nonpolarizable FF used for the MD simulations, which is unable to capture some of the weaker interactions.^51−53^

Although, MD simulations consider the motion of all Li^+^-containing species, the OPLS AA FF underestimates the solvent interactions^54^ as it is a nonpolarizable FF which does not respond to electrostatic changes in the surrounding,^55^ a simplification that often underestimates diffusion coefficients as compared to experimental data.^52^ The PFG-NMR technique, on the other hand, monitors the contributions of ^7^Li and ^19^F nuclei, which means that this technique does not discriminate free cations and anions from ion pairs or aggregates.^18^ PFG-NMR therefore also tends to overestimate , since all Li species are considered (charged or neutral, active or inactive). Second, it measures the self-diffusion of Li^+^ and TFSI driven by internal kinetic energy in the absence of any applied electric field.^56^ This differs from ion migration, which is more relevant for real battery operation.

**Figure 2:**
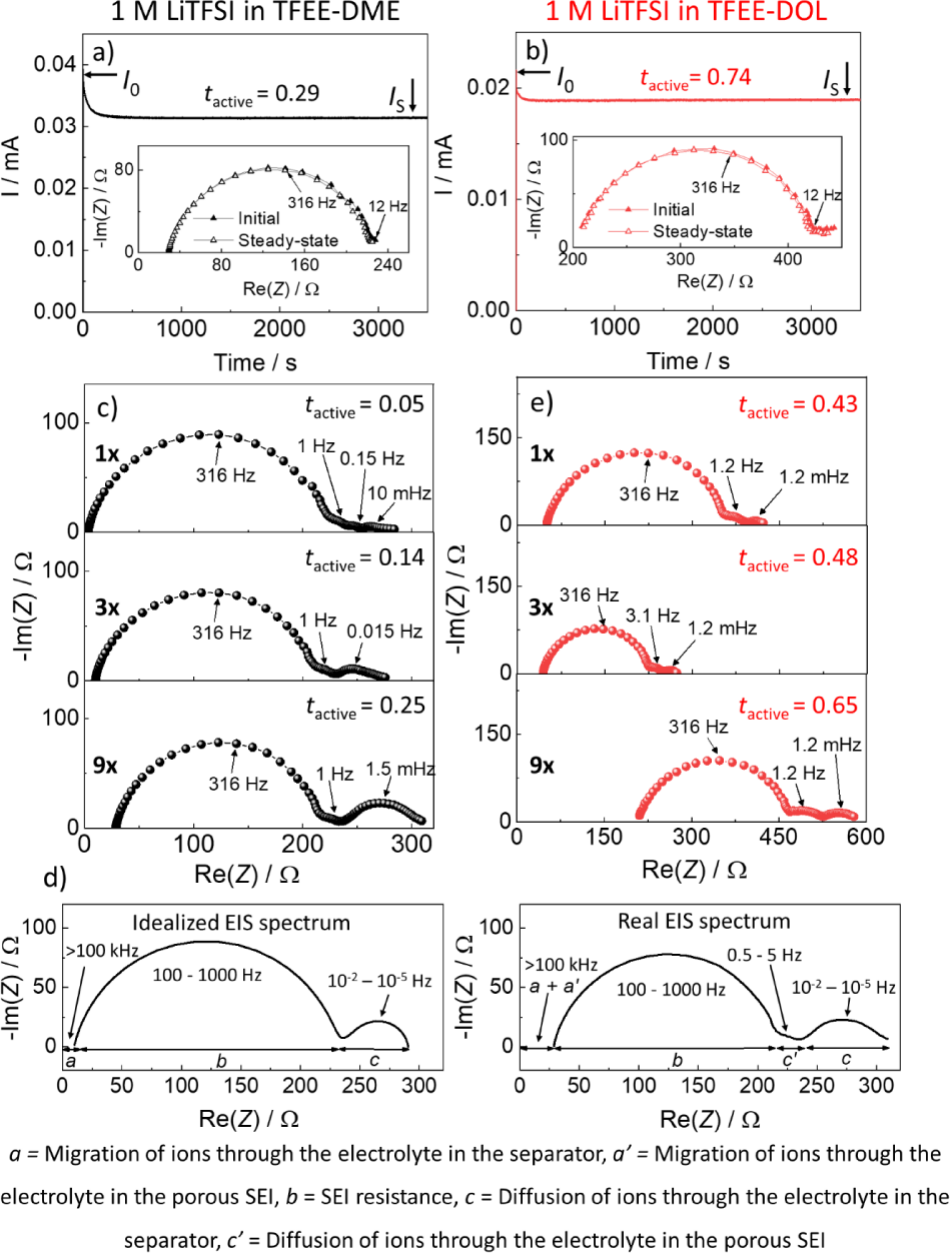
*t*_active_ determination by BV method for (a) 1 M LiTFSI in TFEE-DME and (b) 1 M LiTFSI in TFEE-DOL LHCEs. (c) Low frequency EIS spectra of Li||Li cells with different numbers of separators for 1 M LiTFSI in TFEE-DME LHCE. (d) Scheme showing the difference between idealized and actually measured EIS spectrum of Li metal in contact with electrolyte. In the real EIS spectrum, we have additional contributions coming from the porous SEI layer on Li metal, which is formed due to the interaction of Li with the electrolyte. (e) Low frequency EIS spectra of Li||Li cells with different numbers of separators for 1 M LiTFSI in TFEE-DOL LHCE.

In addition to PFG-NMR spectroscopy and MD simulations, the Bruce-Vincent (BV) method is widely used to determine the  of battery electrolytes.^42,43^ This is an electrochemical method, originally developed for solid polymer electrolytes (SPEs), based on the combination of AC impedance and DC polarization. Although the parameter determined via this method is usually the , in the case of nonideal electrolytes, the measurement actually determines the transport due to all active species in the cell, which we find as more relevant for the present purpose and will term this parameter as *transport number of active ions*, *t*_active_. Here the 1 M LiTFSI in TFEE-DOL electrolyte showed a high *t*_active_ of 0.74 as compared to 0.29 for the 1 M LiTFSI in TFEE-DME electrolyte as shown in Figure 2a,b. The exact values of *I*_o_ and *I*_s_ for each LHCE are mentioned in the zoomed-in polarization curves in Figure S5.

Another technique fundamentally similar to the BV method is the EIS method, which uses low frequency measurements (sub-mHz). The general principle is the same as with the BV method (it gives the value of *t*_active_), except that the diffusional resistance is determined directly from the impedance spectrum and not calculated from the difference between the DC polarization and EIS interfacial and electrolyte resistances. This circumvents the BV method constraints of fast electrode kinetics (Note S1 and Figure S6). The EIS technique may be difficult to apply to *e.g.*, SPEs due to very low peak frequencies of the diffusional contributions but is a viable method for LHCEs. To assess the diffusion at low frequencies, the cells must be stabilized until a steady state is reached (Figures S7 and S8).

The low-frequency part of the spectrum obtained for the 1 M LiTFSI in TFEE-DME electrolyte with one Celgard separator shows three separate contributions (Figure 2c, at 1 Hz, 0.1 Hz, and 1 mHz), suggesting that apart from the idealized EIS contributions several additional processes take place in the cell. One reason could be unwanted interaction of the pouch cell tab with the electrolyte^57^ and a second diffusional impedance due to transport in the porous SEI.^58^ The complexity of the low-frequency EIS region suggests several underlying issues.

First, since the diffusional resistance in the BV method is calculated solely as the difference between the steady state polarization current/voltage and the interfacial arc resistance, this clearly manifests a large disadvantage of the BV method, since one cannot visualize potential interferences due to several low frequency contributions. To more clearly demonstrate the latter issue – Figure 2d shows the idealized EIS spectrum as it is assumed when determining the transport number using the BV method. The impedance spectrum shows three separate contributions: a) the migration of ions in the electrolyte (>100 kHz), b) SEI resistance (100–1000 Hz) and c) diffusion of Li^+^ ions in the electrolyte (10^–2^–10^–5^ Hz). This is relatively close to the reality for SPEs which are often thicker and less conducting than liquid electrolytes (in separators), which results in larger ion migration resistances (*R*_1_ and *R*_2_, see.^58^ Consequently, this means that for SPEs these contributions prevail over any other unwanted interfering impedance contributions. The high frequency resistive intercept and the low frequency Warburg contributions are in those cases indeed mostly due to migration and diffusion of ions in the SPE. In liquid electrolytes, however, the low frequency region of the EIS spectrum is often more complicated (real EIS spectrum in Figure 2d) and contains an additional EIS contribution due to diffusion of ions in the electrolyte contained in the porous SEI layer (*c*’, 0.5–5 Hz.^58^ The occurrence of this low frequency contribution suggests an additional high frequency contribution due to migration of ions in the same porous layer (*a*’, >100 kHz). Since this contribution is hidden in the resistive intercept, calculation of the transport number for solely the separator contribution becomes impossible. The workaround is assuming that the transport number in the electrolyte is contained inside the porous SEI and the porous separator *i.e.*, there are no specific electrolyte – porous structure interactions.

Second, due to low frequency interferences, it is difficult to identify the arc originating from the diffusion of Li^+^ through the separator. One way of identifying the diffusion of Li^+^ ions in the separator is to build cells with increasing the number of separators. Increase from one separator to three or nine for the 1 M LiTFSI in TFEE-DME electrolyte, results in proportional increase of the resistive intercept (*R*_el_) from 3.1 Ω to 9.5 Ω and finally to 28.4 Ω, while the size of the high-frequency arc (at 316 Hz) remains more or less similar (Figures 2c and S9a). The cell with the highest number of separators is expected to have one dominant LF arc for separator diffusion, which is indeed seen at peak frequency of 1.5 mHz. This allows us to determine the expected peak frequencies for separator diffusion even in cells with lower number of separators, since the peak frequency scales inversely with L^2^ (L is the distance between the electrodes).^14,59^ The determined LF arc peak frequencies for 1, 3, and 9 separators are therefore 0.15 Hz > 15 mHz > 1.5 mHz, which is very close to the expected values. The *R*_W_ size changes more or less proportionally to L. Since the error due to additional LF impedance contributions is the smallest in the cell with nine separators added, we assume that the *t*_active_ value calculated from that measurement (0.25, Table S6) is as close to correct as possible.

The same approach was applied to the case of 1 M LiTFSI in TFEE-DOL. This LHCE showed a significantly higher *R*_el_ than 1 M LiTFSI in TFEE-DME LHCE which is consistent with the ionic conductivities measured under ion blocking conditions for these two LHCEs (1.58 *vs* 4.26 mS/cm). Also, surprisingly, expected trends were not observed in this case, *i.e.*, neither *R*_el_ (50.4, 44, and 212 Ω) nor *R*_W_ (68, 48, and 113 Ω) changed proportionally with the monotonic increase in the number of separators (Figure 2e), which we attribute to poor spectra stability due to the continuous rise in the *R*_el_ as shown in Figure S9b. For instance, the Li||Li cell with nine separators and 1 M LiTFSI in TFEE-DME electrolyte showed a stable *R*_el_ for over 24 h, as shown in Figure S10a. In contrast, 1 M LiTFSI in TFEE-DOL showed a significant increase in *R*_el_ from 123 Ω to 192 Ω in 24 h (Figure S10b). This drift indicates a reaction between the electrolyte and Li metal taking place. As discussed earlier,^60^ the resistive intercept of the EIS of Li metal in contact with an electrolyte is expected to have two contributions: the migration resistance of the ions in the separator and in the porous SEI formed on the Li metal in contact with the electrolyte. We hypothesize that the reaction of this electrolyte with the Li metal forms an extensive porous SEI layer, which could explain the drift in the resistive intercept, due to both new layer formation and electrolyte decomposition, and consequently lead to poor spectra stability. We have determined the activation energy of the SEI, *E*_a_(SEI) before and after OCV aging (Figure S11) based on references^61,62^ and found minimal differences both between the electrolytes and before and after aging (Note S2).

**Figure 3:**
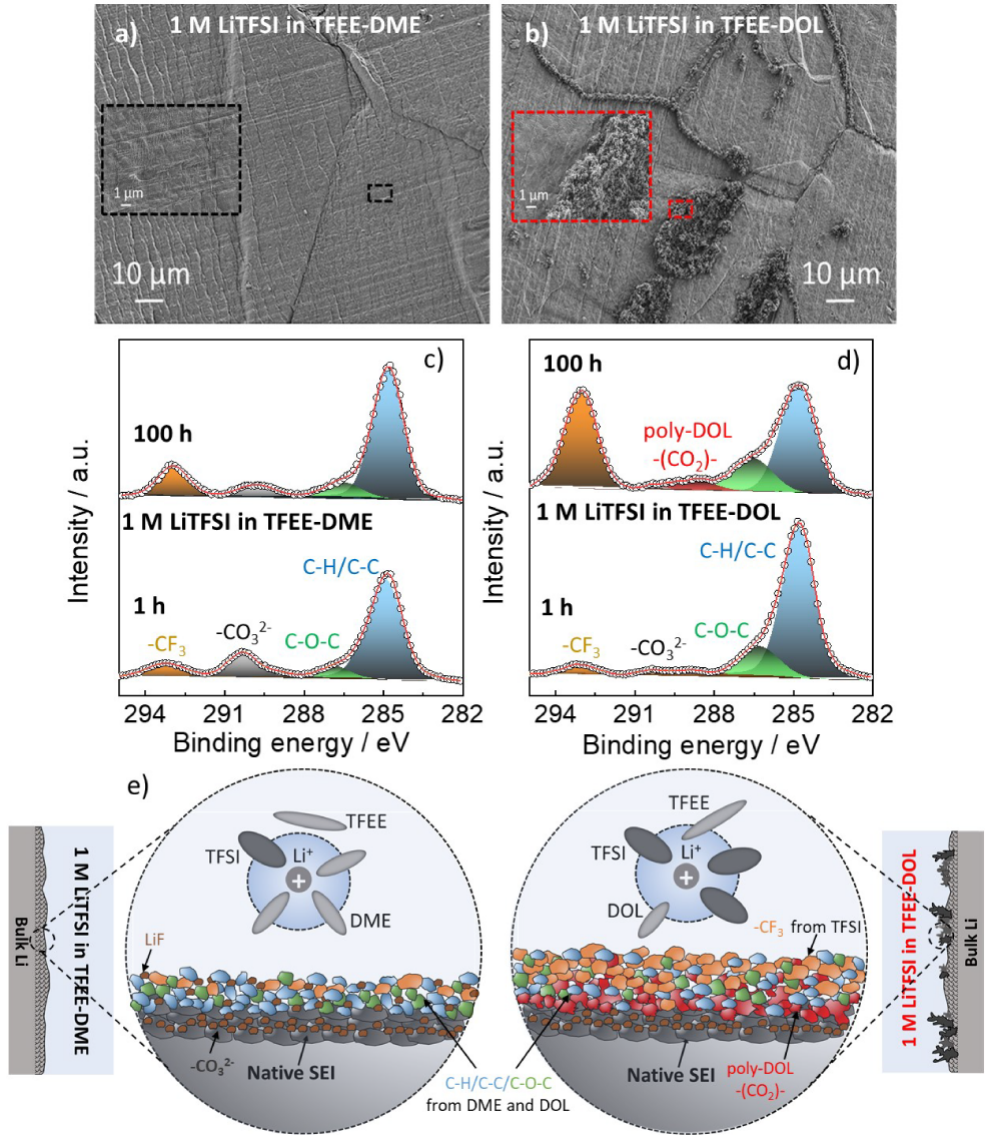
SEM images of Li metal extracted from Li||Li cells for 100 h with (a) 1 M LiTFSI in TFEE-DME and (b) 1 M LiTFSI in TFEE-DOL electrolytes. In the inset of each image, the magnified image of the point of interest is shown. C 1*s* XPS spectra of Li metal treated with (c) 1 M LiTFSI in TFEE-DME and (d) 1 M LiTFSI in TFEE-DOL electrolytes for 1 and 100 h. (e) Schematic illustration of electrolyte solvation and SEIs formed in different electrolytes based on the results shown in this figure, Figure 1, and Note S3. Please note that the schemes presented in (e) are neither exhaustive nor true to scale.

Therefore, this hypothesis was further investigated using *ex situ* SEM and XPS analysis of Li electrodes in contact with the electrolyte. The SEM images of Li metal in contact with 1 M LiTFSI in TFEE-DME showed a flat surface, while Li metal in contact with 1 M LiTFSI in TFEE-DOL electrolyte showed porous formations (Figure 3a,b). We compared Li metal XPS spectra after the electrode had been in contact with the electrolyte for 1 and 100 h. With 1 M LiTFSI in TFEE-DME, there was an increase in −CF_3_ peak and a decrease in the −CO_3_ peak after 100 h (the latter originating from the native SEI components on the bare Li metal surface, Figure S12). This means that the new SEI layer was formed on the top of native SEI of Li metal. The 1 M LiTFSI in TFEE-DOL electrolyte showed the −(CO_2_)– peak after 100 h, indicating that the DOL was decomposed on the Li surface. In addition, Li treated with 1 M LiTFSI in TFEE-DOL electrolyte showed a larger increase of −CF_3_ (Figure 3d). C 1*s* (Figures 3c,d and S13) and F 1*s* (Figure S14). XPS spectra therefore show that the “free” (or less coordinating) DOL from 1 M LiTFSI in TFEE-DOL electrolyte (Figure 1) severely decomposes on Li metal surface and is a possible reason why we observed porous formations on Li metal in Figure 3b. The in-depth discussion of how these electrolytes decompose on Li metal can be found in Note S3, Figures S13 and S14. The solvation structure and its effect on the SEI formation are schematically shown in Figure 3e.

The determination of *t*_active_ in 1 M LiTFSI in the TFEE-DOL case clearly suffers from unwanted interferences due to surface layer formations. We are therefore compelled to estimate an approximate *t*_active_ from the least erroneous measurements. For this purpose, we have chosen to calculate it using both the LF EIS measurement and BV method on the cell with nine separators. Since we know a porous layer is present on the electrode, this determination assumes that *t*_active_ in this porous SEI layer is the same as in the separator, since both migration and diffusional resistances are summed together. In other words, this approach could also be viewed as calculating the “average effective” *t*_active_ in the cell, which is somewhere between 0.65 (EIS) and 0.74 (BV).

The electrolytes therefore have significantly different *t*_active_ with the value for 1 M LiTFSI in TFEE-DME being approximately 1/3 of that for 1 M LiTFSI in TFEE-DOL. We subsequently evaluated the electrochemical performance of the electrolytes to discern if a higher transport number indeed results in improved performance in practice.

**Figure 4:**
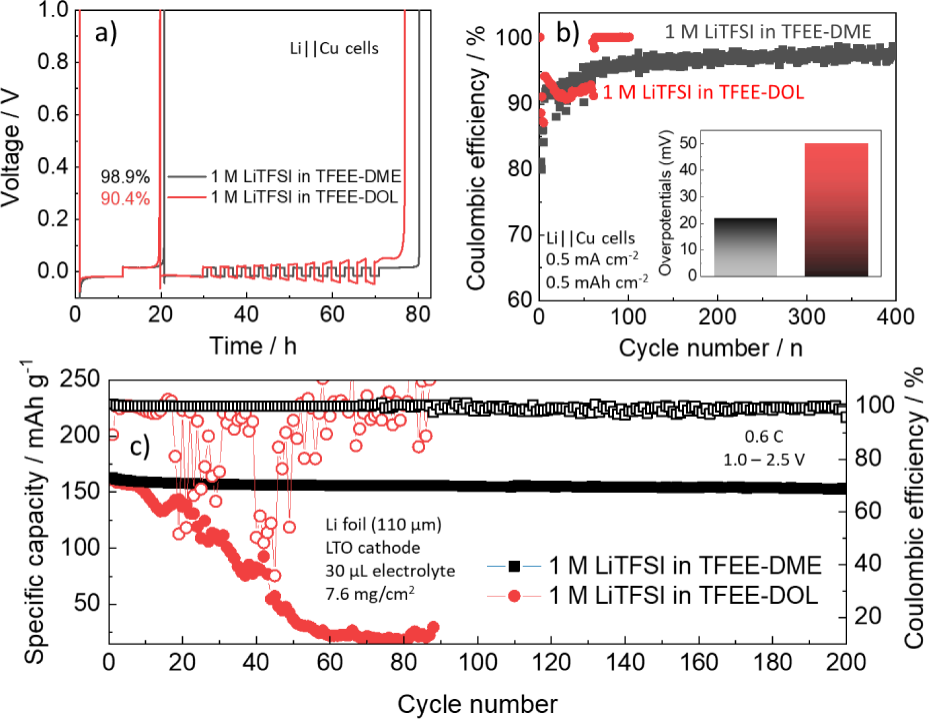
Coulombic efficiencies of Li plating and stripping in Li||Cu cells using the different LHCEs from (a) standard Aurbach method^47^ and (b) galvanostatic cycling. (c) Electrochemical cycling performance of Li||LTO cells with the different LHCEs.

The 1 M LiTFSI in TFEE-DME electrolyte revealed a high CE of 98.9% (Figure 4a), stable for 400 cycles (Figure 4b), and with small overpotentials of *ca*. 21 mV (at 50th cycle, Figure S15a) despite its low *t*_active_, due to its better compatibility with Li metal as shown in Figure 3. The 1 M LiTFSI in TFEE-DOL electrolyte, on the other hand, exhibited low CE (90.4%, Figure 4a), poor stability, and large overpotentials (*ca*. 50 mV at 50th cycle, Figure S15b) (Figure 4b). Electrochemical results consistent with this Li metal performance were also observed for Li||LTO full cells (Figures 4c and 16), depicting that the 1 M LiTFSI in TFEE-DOL electrolyte gives poor electrochemical performance in LMBs regardless of its high *t*_active_. This clearly shows that the high *t*_active_ does not always translate to improved performance with Li metal anodes in practice.

The question now is what *does* then predict the electrolyte performance better? Based on the impedance results shown in Figure 2, we suggest that one should focus on the total impedance (*R*_T_) of Li metal in contact with electrolytes, *i.e.*, the sum of all the migration, interfacial, and diffusional resistances. To gain a detailed understanding, we used *operando* EIS to further validate the importance of measuring *R*_T_ instead of *t*_active_. *Operando* EIS provides information on cell impedance under operation by measuring EIS spectra during cycling. The benefit of this technique is in the fact that EIS data measured at OCV necessitate an equilibration period before the EIS measurement, which can cause significant changes in the cell due to side-reactions such as passive layer formation. The shortcoming of the technique is that the cell potential needs to be stable enough (*i.e.*, changes on the order of a few mV are acceptable) during a single spectrum measurement to ensure EIS spectra stability. This means that spectra measurements need to be relatively fast, which limits the frequency range, so the low frequency information is lost.

**Figure 5:**
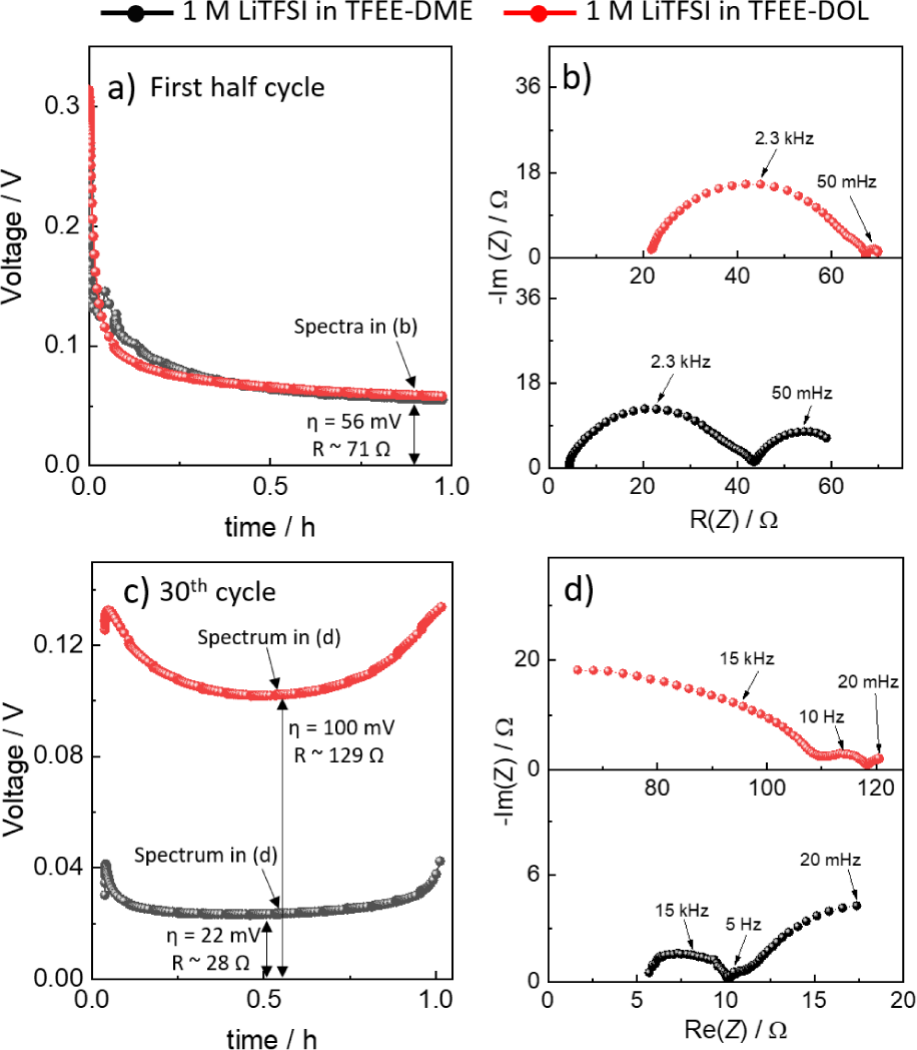
*Operando* EIS measurements on Li||Li cells cycled with the different LHCEs: (a) overpotentials at the first half cycle, (b) GEIS spectra measured during the first half cycle, (c) overpotentials at the 30th cycle, and (d) GEIS spectra measured during 30th cycle.

In the initial cycles, both of the electrolytes showed similar overpotentials of about 60 mV (Figures S17 and 5a). The performance of the electrolytes with significantly different *t*_active_ is therefore at this point identical. *Operando* EIS measurements show that the reason behind this is coincidental. The largest impedance contribution is in both cases the SEI resistance (60% for 1 M LiTFSI in TFEE-DOL and 54% for 1 M LiTFSI in TFEE-DME), while the remainder of *R*_T_ is made up of the electrolyte migration and diffusion resistances in the ratio dictated by the *t*_active_ value (Figure 5b). It just so happens that the sum of resistances for both electrolytes results in the same value. Since the charge transfer reaction of Li metal anode in our system is negligible and the cell operation is mass-transport controlled, the i-η curve is linear at small overpotentials. Thus, assuming linearity, the overpotential can be used to calculate the total resistance, which in this case approximates to 71 Ω. A similar total resistance value can be seen in *operando* EIS spectra (Figure 5b), confirming that the system is close to linearity (Figure 5b).

After 30 cycles of Li plating and stripping, the difference in the overpotentials between these two electrolytes is substantial and again not directly related to the *t*_active_ (22 *vs* 100 mV, Figures 5c and S15). For the 1 M LiTFSI in TFEE-DME electrolyte the *operando* EIS measurement shows a typical impedance spectrum associated with a cycled Li metal electrode.^58^ Specifically, the largest difference is the decrease in the SEI resistance (38 to 5 Ω, Figure 5d), which we associate with Li deposits increasing the surface area of the electrode (Figure S18). The electrolyte migration and diffusion resistances remain in a similar range of values, which results in an increase of their relative contributions. If we extrapolate the impedance spectra to lower frequencies, the total resistance is again similar to the overpotential value of 22 Ω. This value comparison also illustrates why measuring EIS in *operando* mode is paramount—EIS spectra measured at OCV show increased impedance contributions likely stemming from passivation processes during mandatory equilibration period (Figure S19).

For the 1 M LiTFSI in TFEE-DOL electrolyte (Figure 5d), the spectra shape changes significantly with a large high frequency contribution accounting for 84% (∼118 Ω) of the total impedance. We attribute this feature to the sum of the electrolyte resistance and the SEI contribution. The nonconventional shape is therefore due to a large contribution of the migration of ions in the porous part of a thick SEI, which forms on the electrode during cycling.^63^ The characteristic time constant of the process appears to shift with progressive cycling, which moves the corresponding impedance arc (a’ in Figure 2d) to lower frequencies and into the spectral measuring range (in Figure 2 this contribution was a part of the resistive intercept). This results in a drawn-out arc at high frequencies. Decoupling the different contributions in this feature is difficult, since we are *i)* limited to only partial arc determination due to instrumentation limitations and *ii)* because it contains at least three different features. Namely, it consists of migration in separator, migration in porous SEI, and migration in compact SEI, out of which the compact SEI contribution has an additional complication with a 45° feature at high frequency arc start due to the ionic ladder effect.^58^ We therefore have not attempted to determine the individual impedance contributions and only conclude that the porous SEI formation dominates the impedance response of the 1 M LiTFSI in TFEE-DOL electrolyte and therefore also the overpotential and electrode performance. Assuming a 20 kHz peak frequency, 40 Ω resistance, and permittivity of 10, the thickness of the corresponding porous SEI layer is 40 nm (Note S4), which explains why this layer is not evident from SEM micrographs of the cycled electrodes (Figure S18).

The performance of the 1 M LiTFSI in TFEE-DOL electrolyte is therefore poor due to uncontrolled and continuous porous SEI formation processes, while the 1 M LiTFSI in TFEE-DME electrolyte overpotential depends on the degree of high surface area Li deposit formation – a process again governed by the SEI properties. The electrolytes’ cycling performance are thus dictated by the SEI and not by the ion transport through the (bulk) electrolyte, explaining why it cannot be predicted by *t*_active_. Although LHCEs suffer from poor ion transport as reported by Bergstrom et al.,^16^ we suggest that LHCEs are advantageous for LMBs in terms of interfacial stability despite their generally poor ion transport. Furthermore, this study also demonstrates that measuring EIS, particularly under *operando* conditions best describes and correlates to the electrochemical performance of LMBs batteries employing liquid electrolytes.

## Conclusions

We found that our LHCEs based on fluorinated TFEE as diluent have quite complex solvation structures therefore determining  is challenging and still only provides an inaccurate descriptor for the relevant ion transport behavior. In contrast, *t*_active_ (*transport number of active ions)*, describes the portion of charge carried by all ions which react on the electrode surface and we find that EIS gives the most accurate information on any interferences. There are, nevertheless, conflicting results *vs* the conventional paradigm that high *t*_active_ should provide high Coulombic efficiency and stable cycling, that we here are able to connect to significant migration resistance increases upon contact with Li metal. Based on this, we conclude that the interfacial properties at the Li metal anode are more crucial to the performance of LMBs than the ion transport through the (bulk) electrolyte. Therefore, the focus should be on measuring the full impedance spectrum of Li metal with electrolytes (particularly under *operando* conditions) rather than just determining *t*_active_. Not only is its determination difficult due to the inherent complexity of LHCEs, but as shown here, it does not always correlate to the electrolyte performance in practice.

## Supporting Information Available

PFG NMR spectra of electrolytes, Li^+^ solvation structures of electrolytes based on MD simulations, details on the BV method in Note S1, EIS stabilization spectra with the evolution of *R*_el_ with time in different electrolytes, temperature-dependent EIS measurements to calculate *E*_a_(SEI), XPS analysis of Li metal in contact with different electrolytes, Li plating/stripping profiles and overpotentials in Li||Cu cells, charge/discharge profiles of Li||LTO full cells, SEM analysis of cycled Li in different electrolytes, EIS spectra showing the comparison between *operando* and OCV conditions, and calculation of porous layer thickness from the EIS data (PDF)
