# Supplementary material for: Transport Number Determination and Relevance for Lithium Metal Batteries Using Localized Highly Concentrated Electrolytes
Source: Chem Mater. 2025 Mar 17;37(7):2485–95. doi: 10.1021/acs.chemmater.4c03067 (PMC11983712; doi:10.1021/acs.chemmater.4c03067)
Supplement: Supplementary file 1 — cm4c03067_si_001.pdf [file cm4c03067_si_001.pdf]

SUPPORTING INFORMATION

Transport number determination and relevance for lithium metal batteries using localized highly concentrated electrolytes

Hafiz Ahmad Ishfaq[a,b,c], Carolina Cruz Cardona[d], Elena Tchernychova[a], Patrik Johansson[c,d], Miran Gaberšček[a,b], Robert Dominko[a,b,c] and Sara Drvarič Talian[a]

[a] Department of Materials Chemistry, National Institute of Chemistry, Hajdrihova 19, 1000 Ljubljana, Slovenia

[b] Faculty of Chemistry and Chemical Technology, University of Ljubljana, Večna pot 113, 1000 Ljubljana, Slovenia

[c] ALISTORE - European Research Institute, CNRS FR 3104, 15 Rue Baudelocque, Amiens 80039 Cedex, France

[d] Department of Physics, Chalmers University of Technology, 412 96 Gothenburg, Sweden

*Email: sara.drvarictalian@ki.si

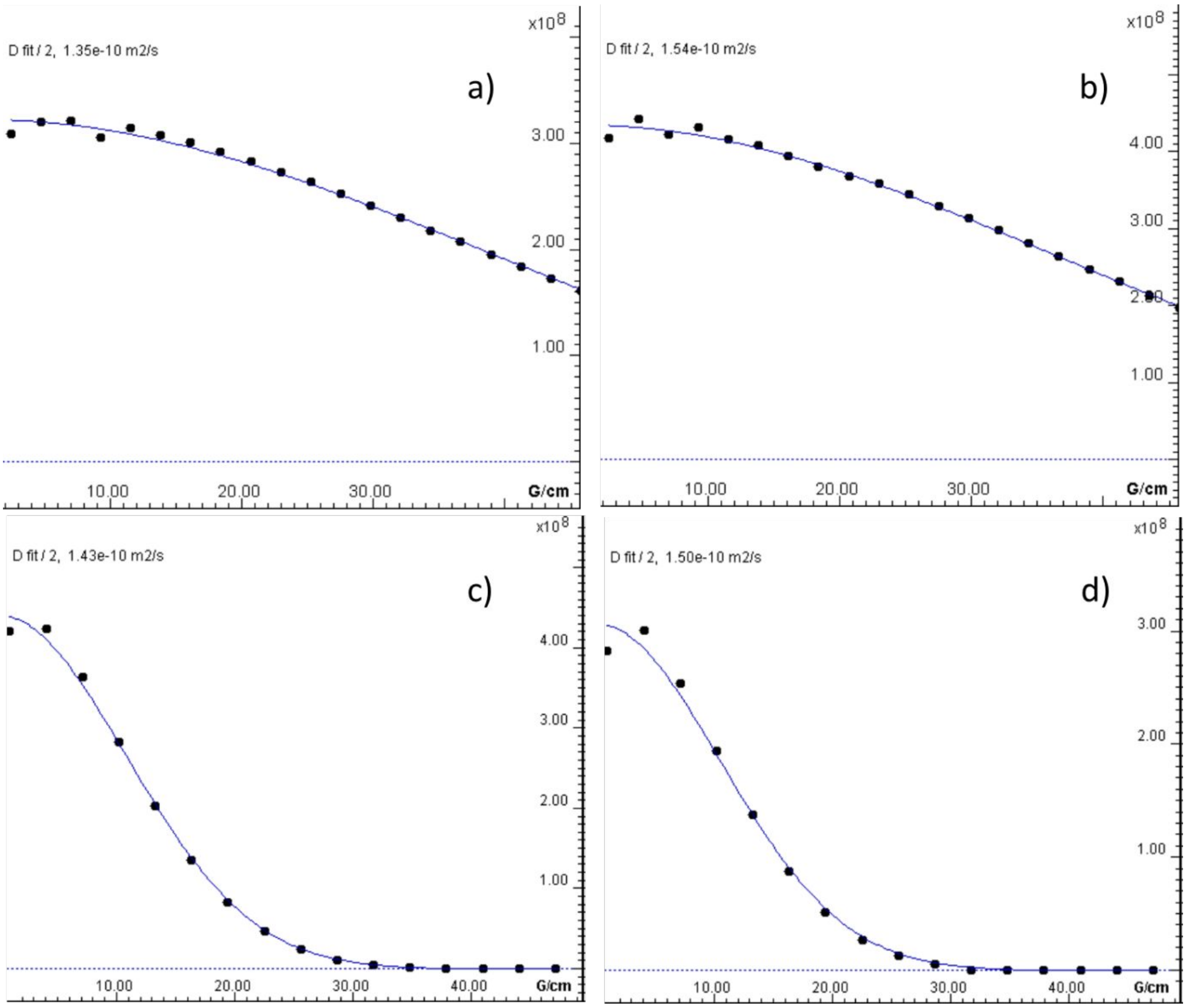

Fig. S1. Li^+ diffusivities for a) 1 M LiTFSI in TFEE-DME and (b)1 M LiTFSI in TFEE-DOL electrolytes calculated from ^{7}Li PFG-NMR spectra. TFSI diffusivities for (c) 1 M LiTFSI in TFEE-DME and (d) 1 M LiTFSI in TFEE-DOL electrolytes calculated from ^{19}F PFG-NMR spectra. All the calculations were made by fitting PFG-NMR with the Stejskal-Tanner equation[1] using Bruker Dynamics Center.

Table S1. Values for the different parameters used in the Stejskal-Tanner equation[1] to get PFG-NMR spectra.

PFG-NMR	γ (rad/s gauss)	δ (s)	Δ (s)	g (G/cm)
^{7}Li	10396	0.0015	0.99	variable
^{19}F	25165	0.004	0.3	variable

Table S2. Number of solvent molecules and ions used in cubic MD simulation boxes.

Electrolyte	# molecules - solvent 1	# molecules - solvent 2	#Li^+	# TFSI
1 M LiTFSI in TFEE-DME	490 TFEE	836 DME	87	87
1 M LiTFSI in TFEE-DOL	386 TFEE	978 DOL	68	68

Table S3. Coordination number and radial distances of different pairs in different electrolytes calculated by MD simulations.

Pair	1 M LiTFSI in TFEE-DME		1 M LiTFSI in TFEE-DOL	
	Coordination	Radial distance (Å)	Coordination	Radial distance (Å)
Li^+-O (TFSI)	0.48	3.37	3.78	3.28
Li^+-O (DOL)	---	---	0.54	3.44
Li^+-O (DME)	2.72	4.02	---	---
Li^+-O (TFEE)	---	---	0.1	3.81

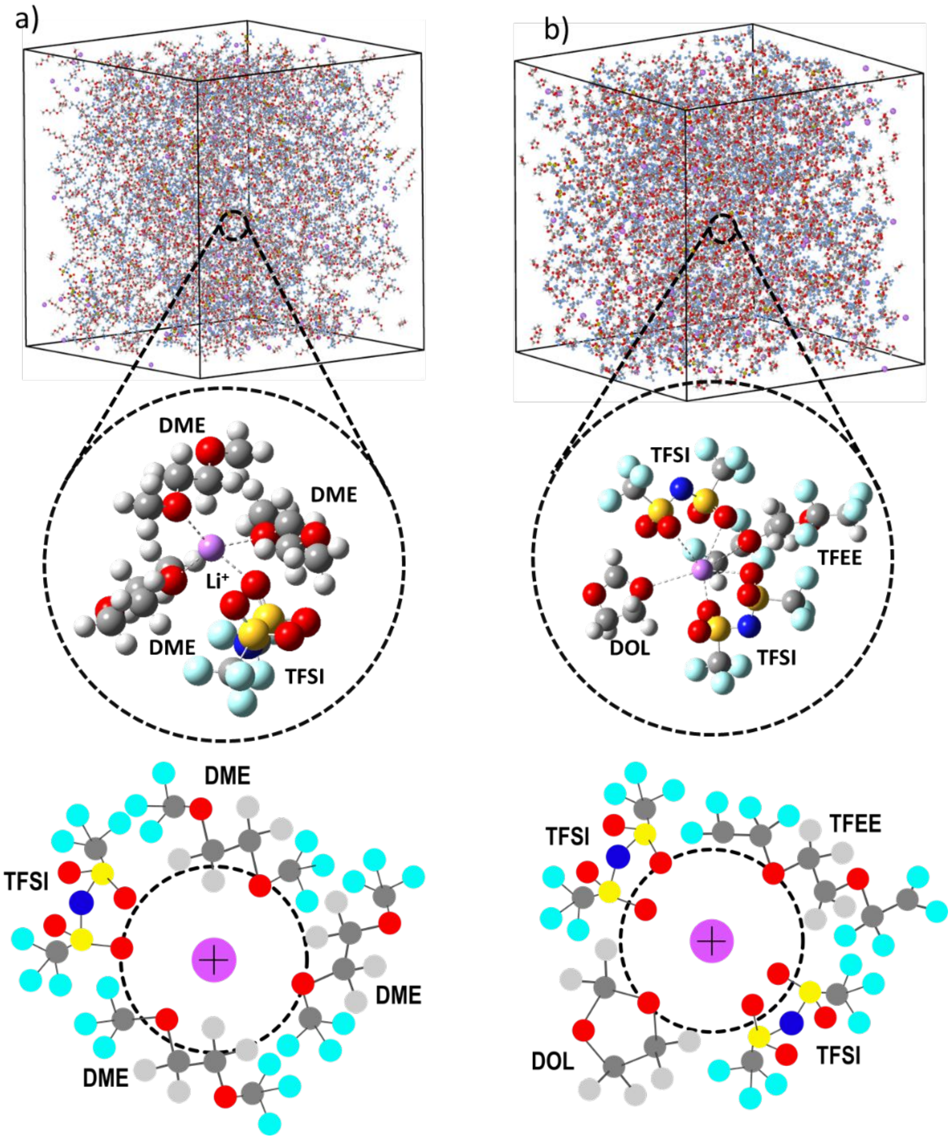

Fig. S2. Li^+ solvation structures of (a) 1 M LiTFSI in TFEE-DME and (b) 1 M LiTFSI in TFEE-DOL electrolytes based on MD simulations.

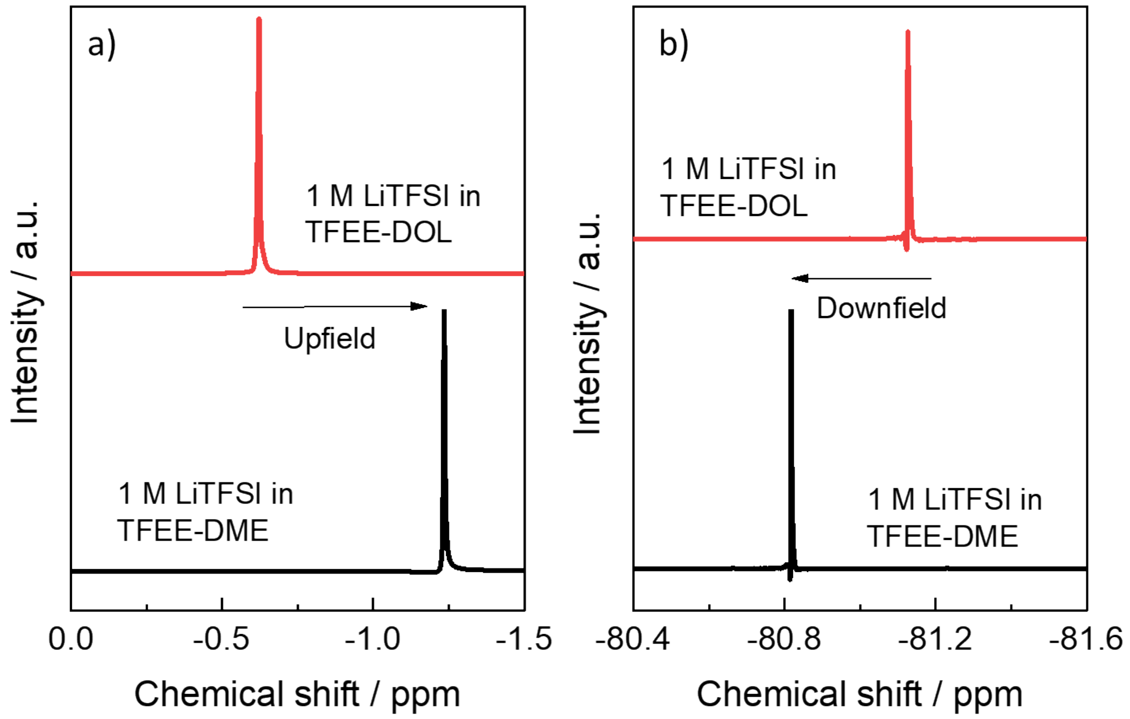

Fig. S3. (a) 7Li and (b) 19F NMR spectra of different electrolytes.

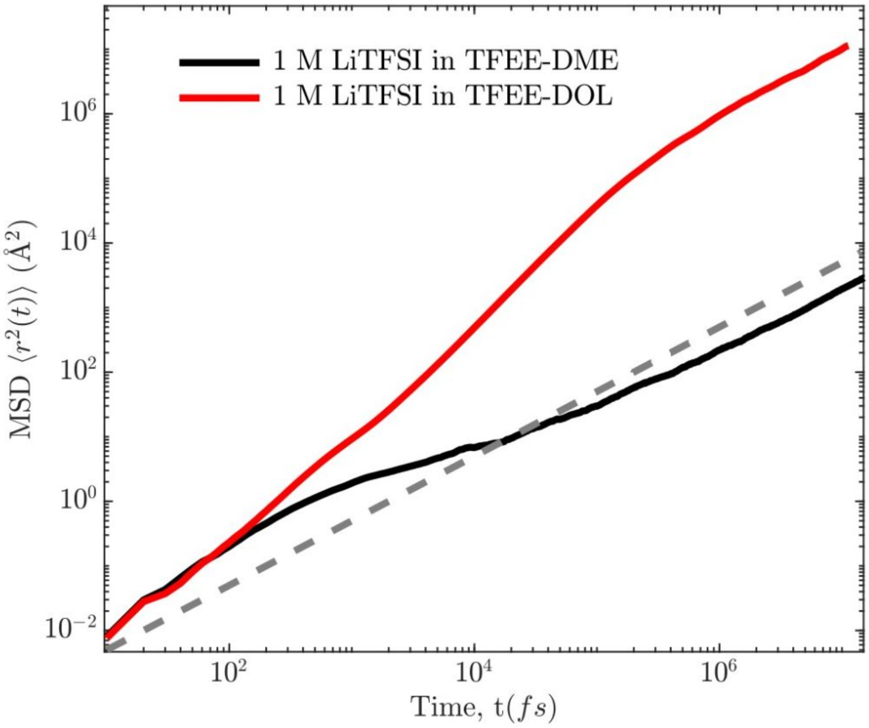

Fig. S4. MSD as a function of time for different electrolytes. The plot provides insight into the mobility of Li^+ in the electrolytes by illustrating how the MSD evolves.

Table S4. Li^+ and TFSI diffusivities obtained from MD simulations for different electrolytes and their respective t_{Li^+} values.

**Electrolytes**	**D_{Li+} (m^2/s)**	**D_{TFSI} (m^2/s)**	**t_{Li+}**
1 M LiTFSI in TFEE-DME	$4.06 \cdot 10^{-11}$	$4.95 \cdot 10^{-11}$	0.45
1 M LiTFSI in TFEE-DOL	$4.16 \cdot 10^{-8}$	$4.17 \cdot 10^{-8}$	0.50

Table S5. Li^+ and TFSI diffusivities obtained from NMR for different electrolytes and their respective t_{Li^+} values.

**Electrolytes**	**D_{Li+} (m^2/s)**	**D_{TFSI} (m^2/s)**	**t_{Li+}**
1 M LiTFSI in TFEE-DME	$1.32 \cdot 10^{-10}$	$1.43 \cdot 10^{-10}$	0.48
1 M LiTFSI in TFEE-DOL	$1.54 \cdot 10^{-10}$	$1.50 \cdot 10^{-10}$	0.51

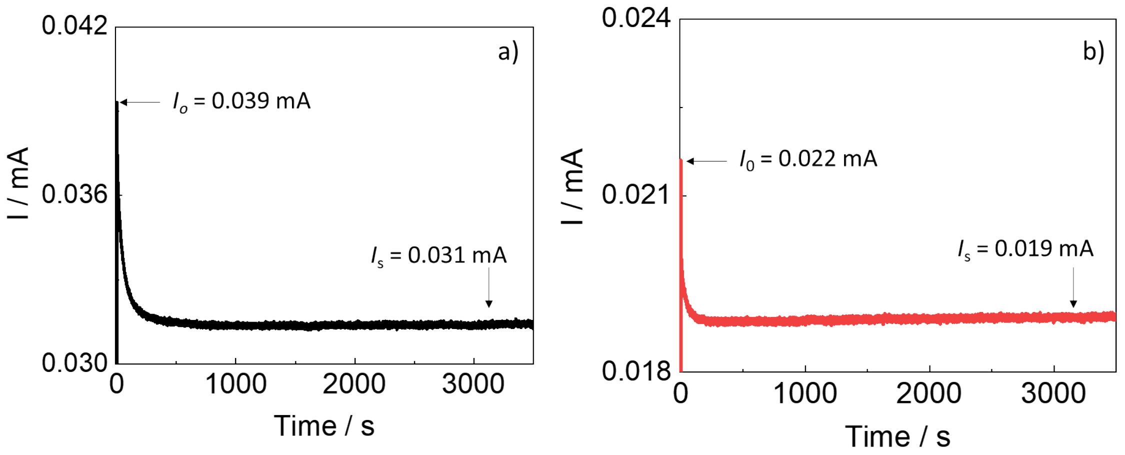

Fig. S5. Potentiostatic polarization curves at ΔV of 10 mV for; (a) 1 M LiTFSI in TFEE-DME and (b) 1 M LiTFSI in TFEE-DOL electrolyte.

Note 1:

The calculation of the transport number from the BV method assumes that the electrode has fast kinetics, so that the resistance calculated from the Ohm's law can be directly compared to the resistances determined from the EIS spectra. The resistance calculated by Ohm's law from the overpotential and current (termed as R_{total} on **Fig. S6a**) is the slope between zero and the point corresponding to the overpotential applied to the cell on the current-voltage characteristic curve (**Fig. S6c**). The total resistance at zero frequency (*i.e.* the sum of all resistances in the EIS spectrum, R_0 on **Fig. S6b**) is, on the other hand, the slope of the curve in the narrow window next to the relevant overpotential (this latter narrow window corresponds to the AC amplitude applied during the impedance spectroscopy measurement, **Fig. S6d**). If we assume an electrode has a non-negligible charge transfer contribution, then its current-voltage characteristic is not linear, why one cannot calculate the diffusion contribution as suggested in the BV method, since we cannot directly equate R_{total} and R_0.

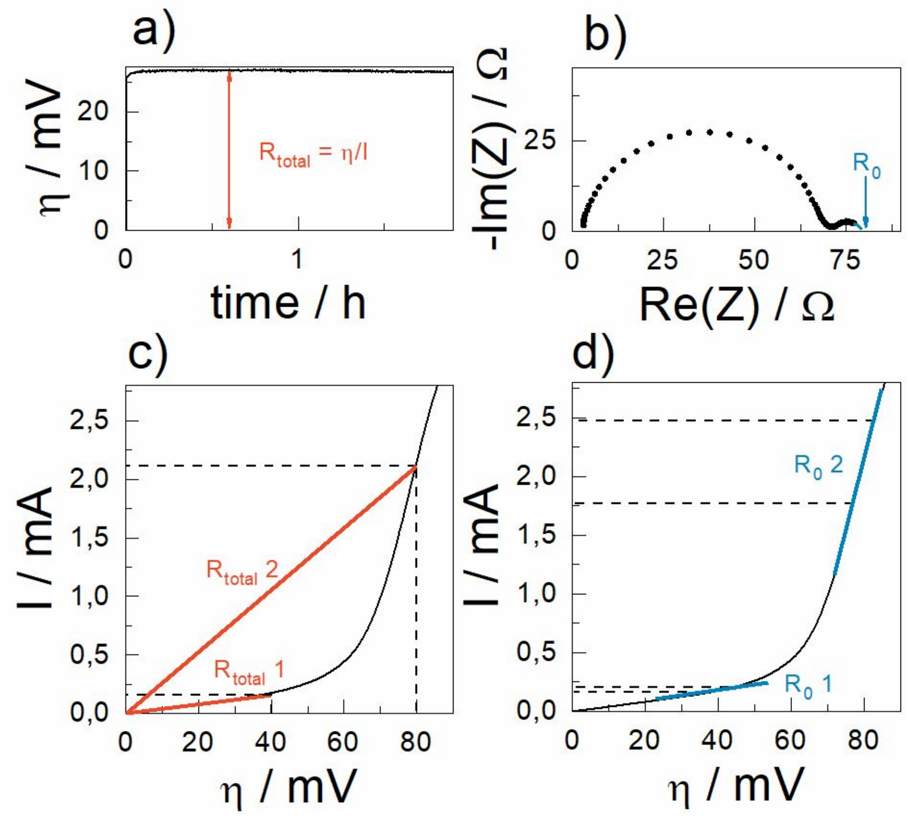

Fig. S6: Schematic representation of the difference between the sum of relevant resistive parts of all impedance contributions, R_0, and total resistance, R_{total}, calculated from the current imposed and overpotential value measured. Schematic representation of the value of a) R_{total} and b) R_0. c) Values of R_{total} at different points of the current vs. potential curve and d) values of R_0 at different points of the current vs. potential curve. Note that the values of R_{total} 1 and R_0 1 are close because the current vs. potential curve is linear in that range. R_{total} 2 and R_0 2, on the other hand, significantly differ."

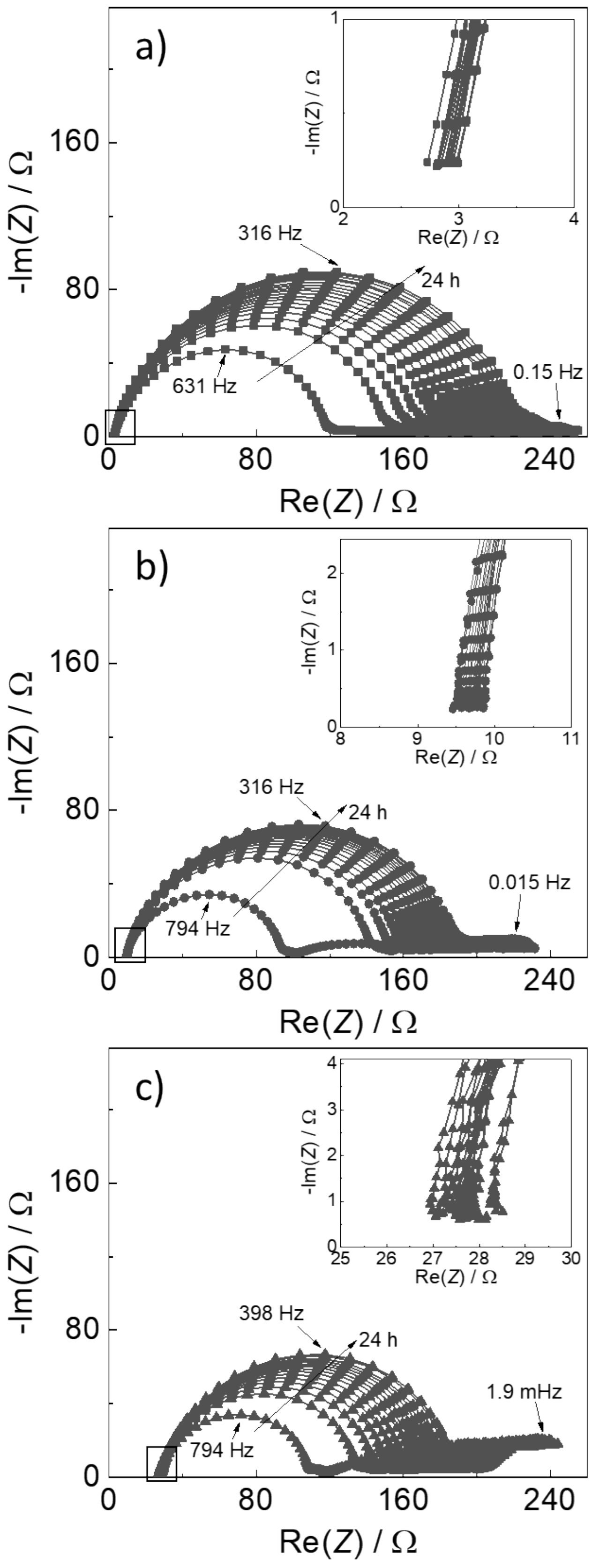

Fig. S7. EIS spectra recorded on Li||Li cells with 1 M LiTFSI in TFEE-DME electrolyte during ~24 hours of stabilization with (a) one, (b) three, and (c) nine Celgard separators. The Inset in each graph showed the magnified R_{el} part of the impedance spectra.

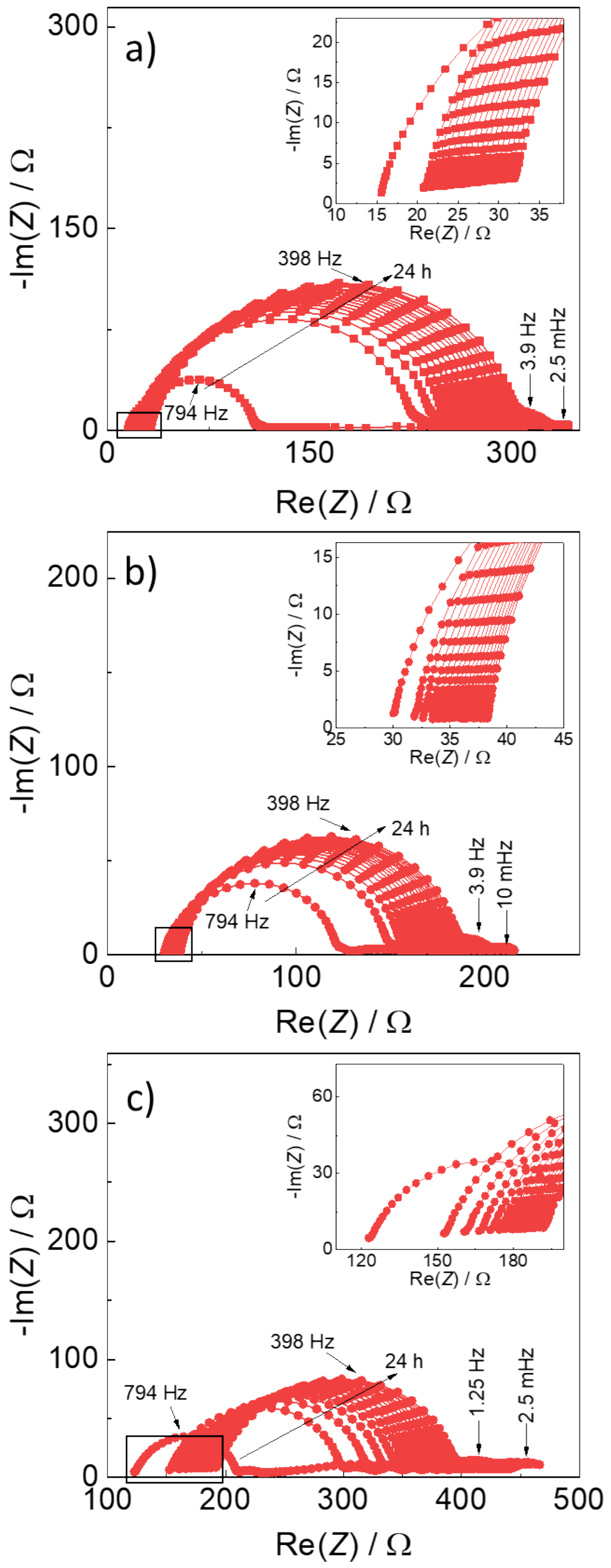

Fig. S8. EIS spectra recorded on Li||Li cells with 1 M LiTFSI in TFEE-DOL electrolyte during ~24 hours of stabilization with (a) one, (b) three, and (c) nine Celgard separators. The Inset of each graph showed the magnified R_{el} part of the impedance spectra.

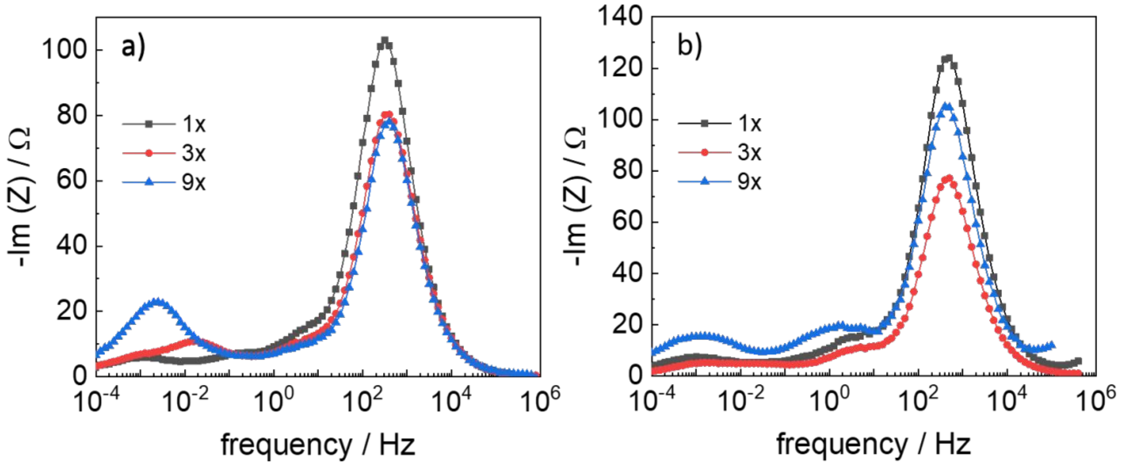

Fig. S9. Bode plots for stabilized Li||Li cells with different number of Celgard separators measured in the frequency range of 1 MHz to 0.1 mHz for (a) 1 M LiTFSI in TFEE-DME and (b) 1 M LiTFSI in TFEE-DOL electrolyte.

Table S6. t_{active} values calculated by EIS and BV method with different separators in Li||Li cells.

	t_{active} for different electrolytes			
Number of separators	1 M LiTFSI in TFEE-DME		1 M LiTFSI in TFEE-DOL	
	By EIS	By BV	By EIS	By BV
1x	0.057	0.14	0.43	0.65
3x	0.14	0.21	0.48	0.67
9x	0.25	0.29	0.65	0.74

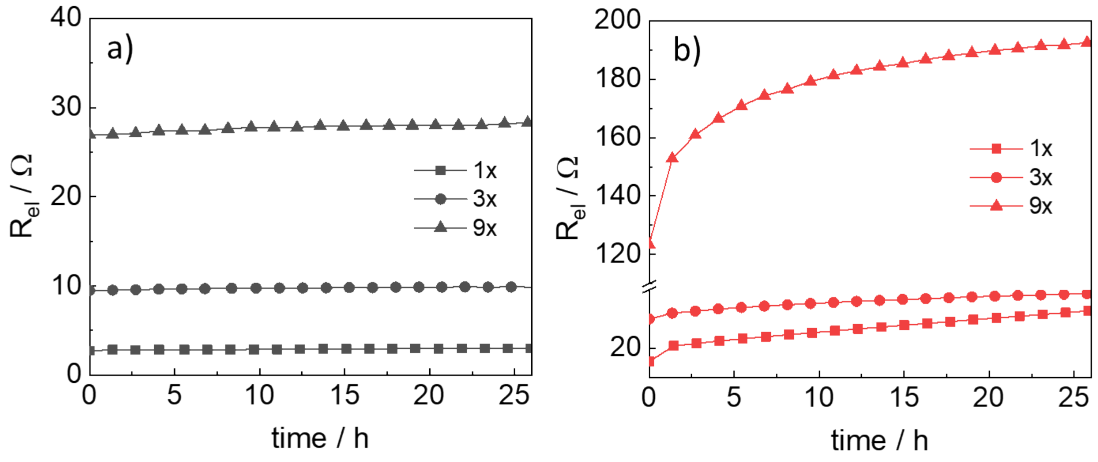

Fig. S10. Change of R_{el} (resistive intercept of EIS spectra at 316.2 kHz extracted from **Fig. S4** and **Fig. S5**) with time for (a) 1 M LiTFSI in TFEE-DME and (b) 1 M LiTFSI in TFEE-DOL electrolytes.

Note 2: Temperature-dependent EIS measurements and E_a(SEI)

Based on the literature[2,3], we investigated long-term SEI growth analysis and have calculated the E_a(SEI) of Li metal in contact with 1 M LiTFSI in TFEE-DME and 1 M LiTFSI in TFEE-DOL LHCEs. For this purpose, the symmetric Li||Li cells were assembled and then subjected to OCV aging for approximately 30 and 500 h, during which the R_{SEI} was monitored. After the subsequent aging, temperature-dependent EIS measurements with 10 mV amplitude in the range from 5 °C to 45 °C were performed. The results are shown in **Fig. S11**. The 1 M LiTFSI in TFEE-DME LHCEs showed an E_α(SEI) of 0.68 eV after 30 h of OCV aging, which did not change significantly (0.69 eV) for about 500 h after SEI growth, as shown in **Fig. S11a and S11c**. The TFEE-DOL LHCEs in contact with Li metal also showed no change in E_a(SEI) after 500 h of OCV aging (**Fig. S11b and S11d, 0.75 eV and 0.72 eV**). The difference between E_a(SEI) for these electrolytes is also small, indicating that the level of SEI compactness is almost the same in both cases. This is likely because of the physicochemical origin of the SEI arc, which was used to calculate the E_a(SEI) in the references[2,3]. It corresponds to the compact SEI, through which the transport of lithium takes place in the form of migration of Li^+ in the solid material of the SEI. The E_a(SEI) measure describes the properties of this solid material. What we are discussing in this study in relation to the properties of the porous SEI is in relation to the cell layer where transport of Li^+ ions takes place as movement in the electrolyte inside the SEI pores (*i.e.* migration and diffusion). This transport is not seen in EIS spectra as part of the arc at 100-1000 Hz, but as two separate contributions – high frequency migration contribution (part of the resistive intercept) and low frequency diffusion contribution (0.5-5 Hz)[4,5]. Determining how the "compactness" of the compact SEI changes does not directly correlate with the contribution of the transport in the electrolyte contained in porous SEI.

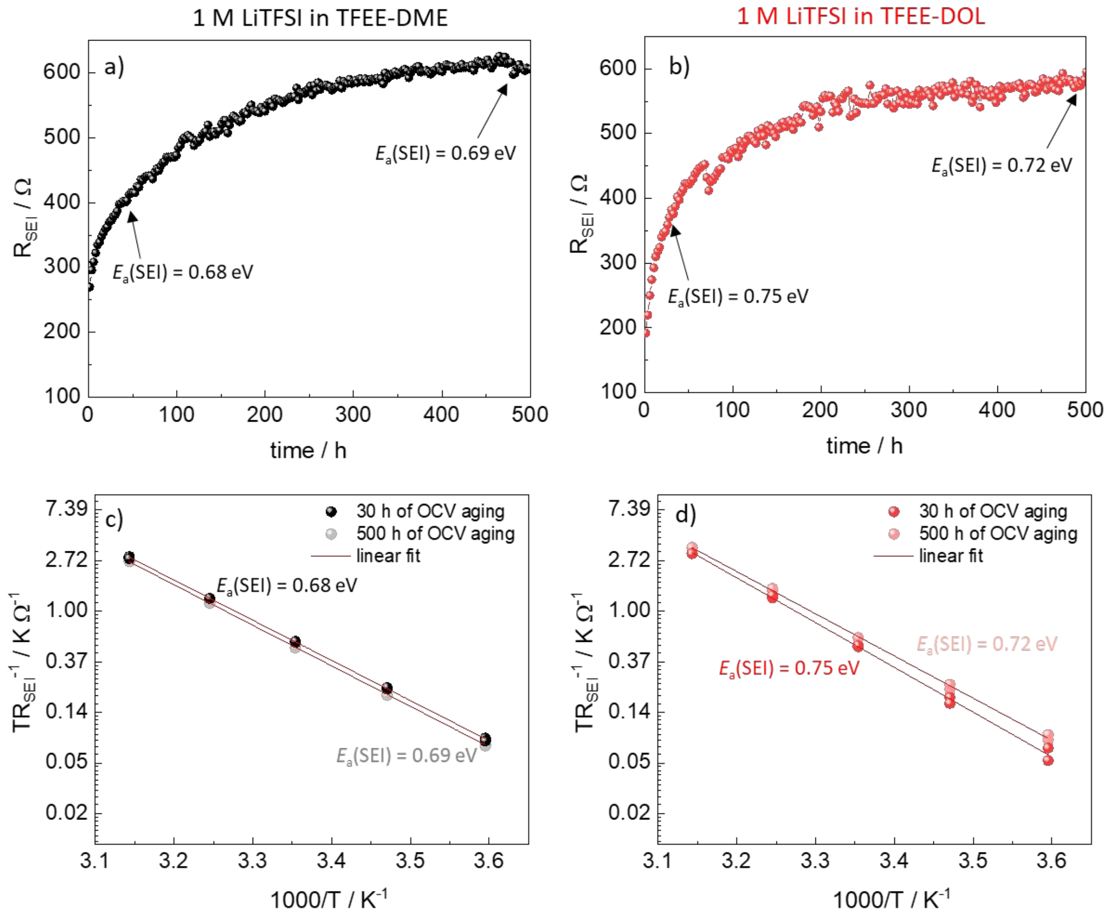

Fig. S11. Time dependent R_{SEI} measured in Li||Li cells with (a) 1 M LiTFSI in TFEE-DME and (b) 1 M LiTFSI in TFEE-DOL LHCEs stored at OCV for 500 h. Arrhenius plots and the E_a of the Li||Li cells with (c) 1 M LiTFSI in TFEE-DME and (d) 1 M LiTFSI in TFEE-DOL LHCEs after 30 and 500 h of OCV aging.

Note 3: XPS analysis of Li in contact with electrolyte

Table S7. Attribution of species to the BE revealed by XPS.

Detail spectrum	Binding energy [eV]	Attributed species	Assumed origin	Ref
C 1*s*	284.8	C-C/C-H	DOL/DME solvent residuals or decomposition products, (alcoholates) aliphatic carbons, alkoxides, or carboxylates	6,7
	286.5	C-O-O	DOL/DME solvent residuals or decomposition products, alkoxides, or carboxylates	7,8
	288	$-(CO_2)-$	DOL decomposition product formed poly-DOL or carbonyl or polymeric species	7,9,10
	289.9	CO_3^{-2}	Carbonates, *e.g.* Li_2CO_3 or semi-carbonates as inorganic salt components present in native SEI	7,10–12
	292.8	$-CF_3$	LiTFSI conducting salt	13
F 1*s*	684.9	LiF	LiTFSI decomposition product	13
	688.7	$-CF_3$	LiTFSI conducting salt or its decomposition	13

The C 1*s* signal shown in **Fig. 3c and 3d** can be divided into five different peaks, each corresponding to a different chemical species (**Table S7**). The highest intensity of the C 1*s* signal for the Li treated in both electrolytes is observed at a binding energy of 284.8 eV, which can be attributed to C-H or C-C species. In addition, a peak at 286.5 eV is assigned to C-O bonds in the R_3CO-R.[7,8] These two peaks are the decomposition products of DOL and DME solvents in the electrolytes. The component detected at about 287.9 eV is not present in the 1 M LiTFSI in TFEE-DME sample and is solely attributed to carbonyl or polymer species $(CH_2CH_2OCH_2)_n$ resulting from the decomposition of DOL in the 1 M LiTFSI in TFEE-DOL electrolytes.[7,9,10] We identify carbonates and/or semicarbonates (CO_3^{2-}, 289.9 eV) present in all samples. These components are known to form SEI on the bare Li metal surface[7,10–12] and are also observed in the native SEI on bare Li metal (**Fig. S12**). The additional line at 292.9 eV represents the $-CF_3$ group in LiTFSI and is observed in all samples, likely originating either from the residual conducting salt or from the decomposition of LiTFSI.[13] Two different peaks were observed in F 1*s* spectra (**Fig. S14**): one at 684.9 eV and 688.6 eV attributed to LiF and $-CF_3$, respectively.[13]

We performed the depth profile analysis using XPS to further analyze the Li metal surface treated with these electrolytes for 100 hours. An increase in the $-(CO_2)-$ peak (colored red in **Fig. S11 b**) was observed until 60 seconds after sputtering, after which it began to decrease. This suggests that the DOL from 1 M LiTFSI in TFEE-DOL electrolyte reacts with Li metal. The peak intensity of the C 1*s* spectra of Li treated with 1 M LiTFSI in TFEE-DME decreased with sputtering time, indicating less decomposition of the solvent (DME) in this electrolyte. In addition, the F 1*s* spectra shown in **Fig. S12**

a and c revealed a relatively high presence of LiF on the surface of Li metal treated in 1 M LiTFSI in TFEE-DME electrolyte compared to Li treated in 1 M LiTFSI in TFEE-DOL electrolyte (**Fig. S12 b and d**). In essence, based on the XPS and SEM results, we observed that 1 M LiTFSI in TFEE-DOL electrolyte decomposed more in comparison to the 1 M LiTFSI in TFEE-DME electrolyte. This explains the reason behind the continuous increase in R_{el} (**Fig. S9b**) and thus explains the poor EIS spectra stability presented in **Fig. 2b**. Moreover, the lower decomposition of 1 M LiTFSI in TFEE-DME electrolyte and the presence of LiF on the Li metal explains why the porous formations in **Fig. 3a** were not observed, thus explaining its R_{el} stability.

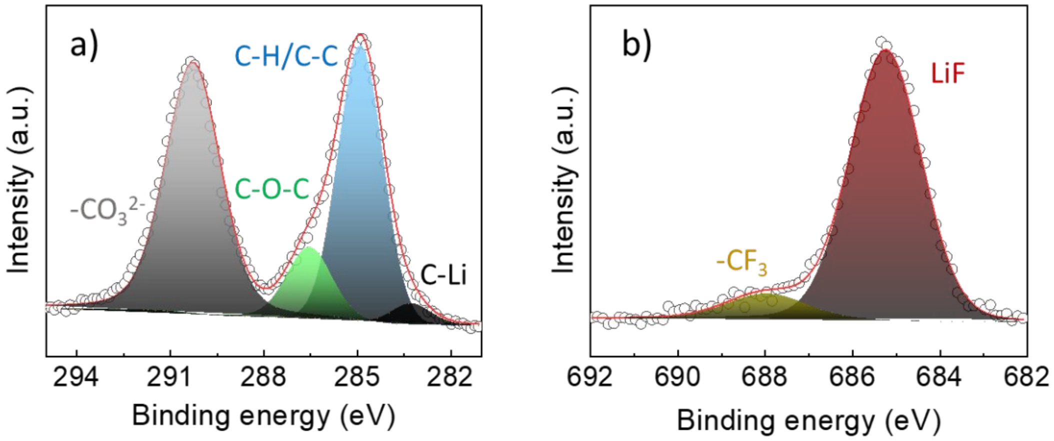

Fig. S12. a) C 1*s* and b) F 1*s* spectra of bare Li metal.

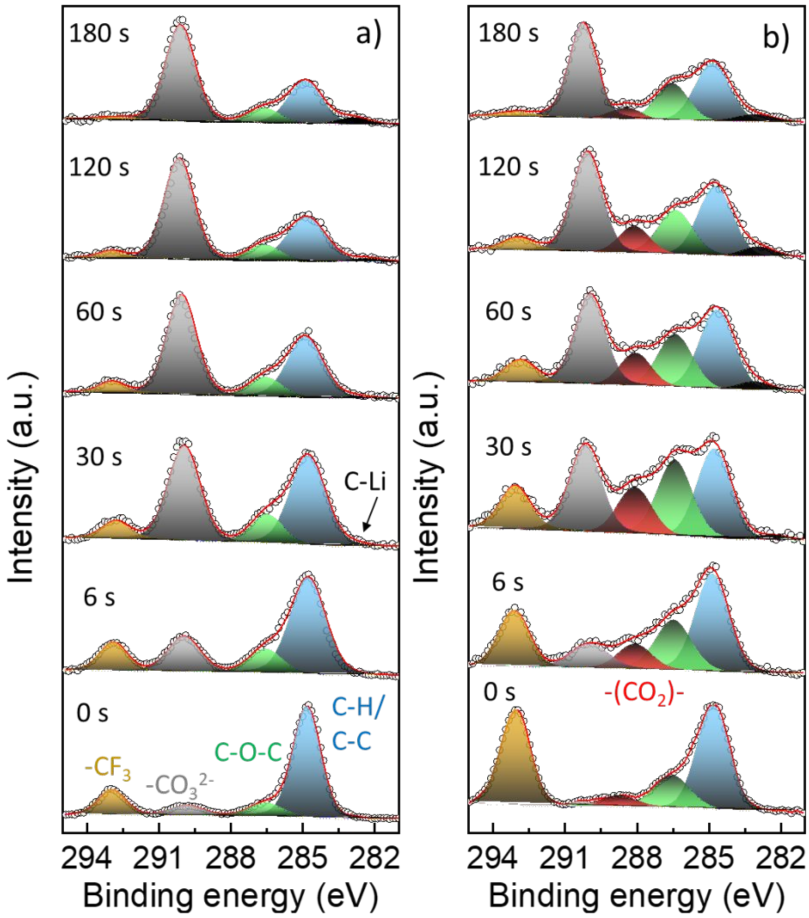

Fig. S13. C 1*s* XPS spectra for depth profiling of Li metal treated with (e) 1 M LiTFSI in TFEE-DME and (f) 1 M LiTFSI in TFEE-DOL electrolytes for 100 hours.

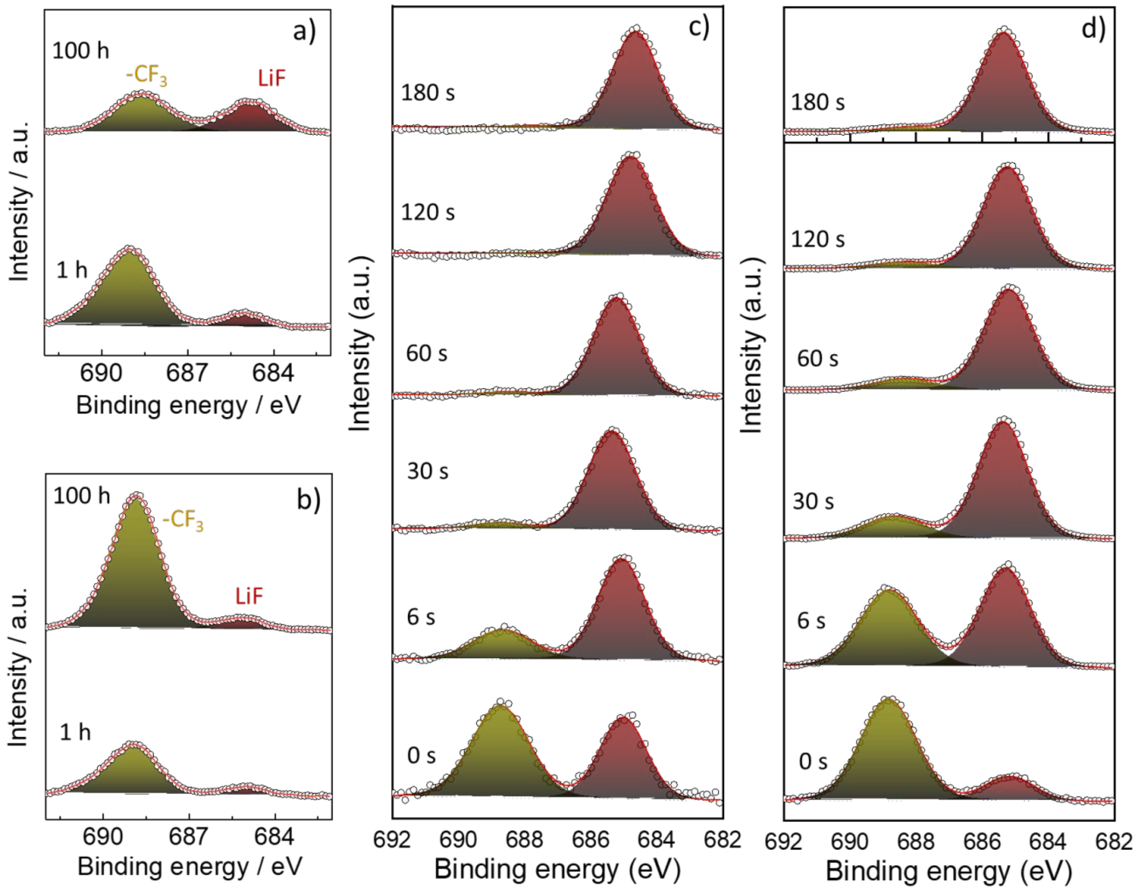

Fig. S14. F 1*s* XPS spectra of Li metal treated with (a) 1 M LiTFSI in TFEE-DME and (b) 1 M LiTFSI in TFEE-DOL electrolytes for 1 and 100 hours. F 1s XPS spectra for depth profiling of Li metal treated with (c) 1 M LiTFSI in TFEE-DME and (d) 1 M LiTFSI in TFEE-DOL electrolytes for 100 hours.

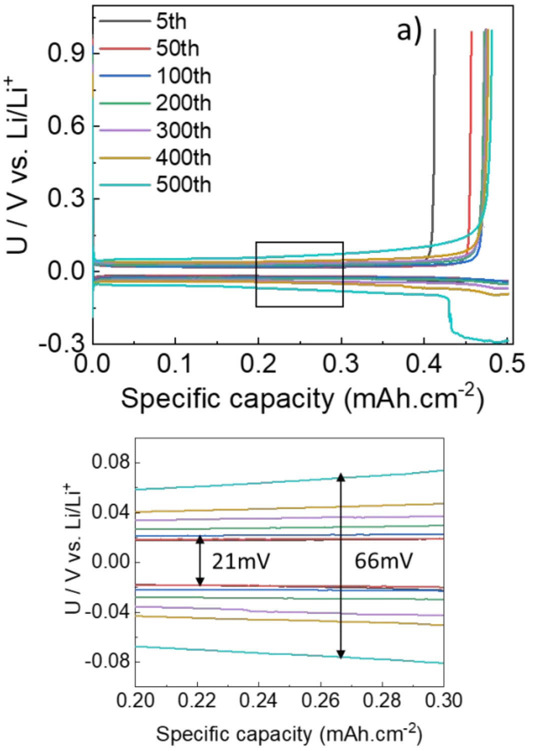

Fig. S15. Li plating and stripping profiles and overpotentials of Li||Cu half cells tested at a current density of 0.5 mA cm^{-2} with a cutoff capacity of 0.5 mAh cm^{-2} using: (a) 1 M LiTFSI in TFEE-DME and (b) 1 M LiTFSI in TFEE-DOL electrolytes.

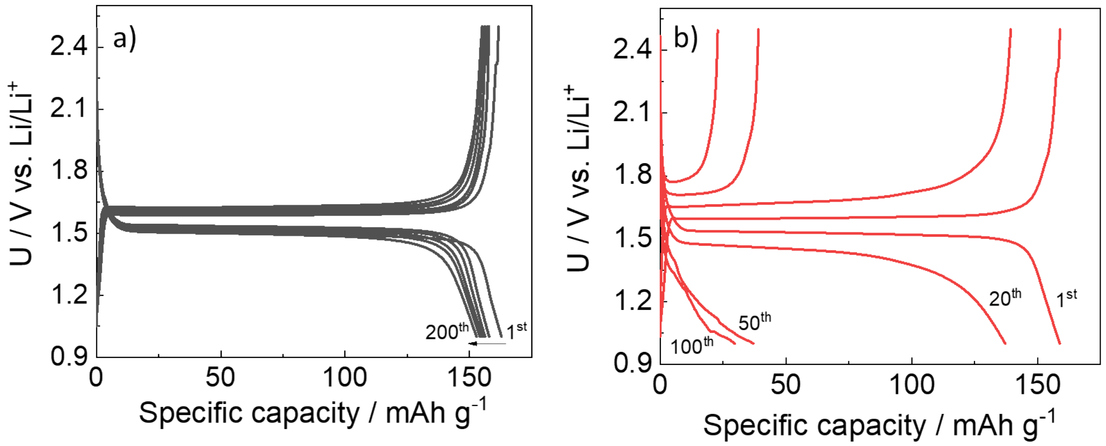

Fig. S16. Charge–discharge profiles of Li||LTO full cells cycled at 0.6 C using: (a) 1 M LiTFSI in TFEE-DME and (b) 1 M LiTFSI in TFEE-DOL electrolytes.

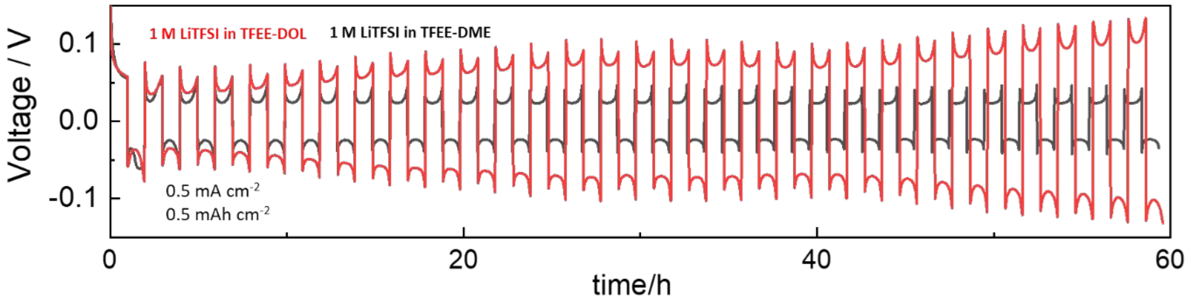

Fig. S17. Li||Li symmetric cells cycled at a current density of 0.5 mA cm^{-2} to a cutoff capacity of 0.5mAh cm^{-2} using the different electrolytes.

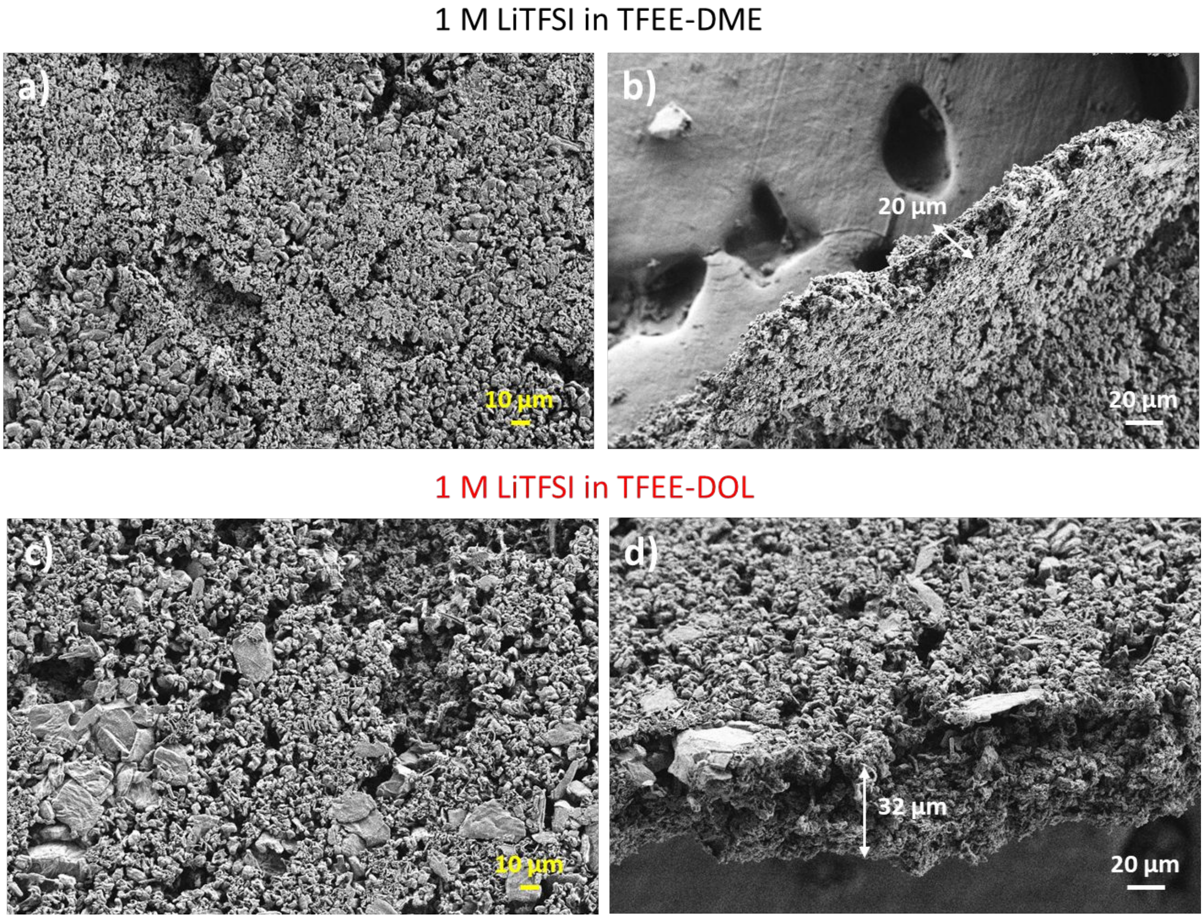

Fig. S18. SEM of Li metal extracted from Li||Li cells cycled for 30 cycles at a current density of 0.5 mA cm^{-2} to a cutoff capacity of 0.5mAh cm^{-2} using (a and b) 1 M LiTFSI in TFEE-DME and (c and d) 1 M LiTFSI in TFEE-DOL electrolytes.

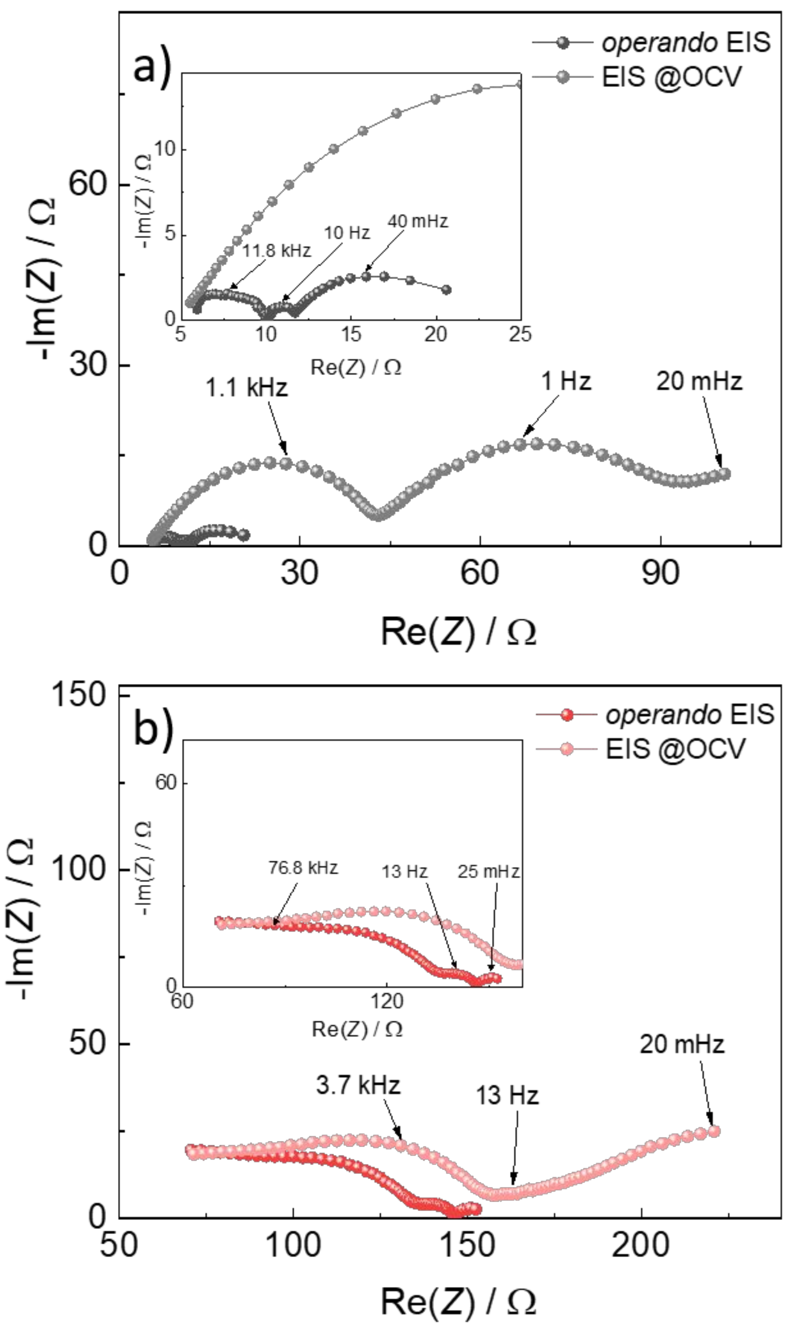

Fig. S19. Comparison between *operando* EIS and EIS at OCV measured in Li||Li cells with (a) 1 M LiTFSI in TFEE-DME and (b) 1 M LiTFSI in TFEE-DOL electrolytes.

Note 4: Calculation of porous layer thickness from EIS data

The thickness (*l*) of the porous layer for the EIS peak was calculated with the following data:

$\nu_{peak} = 20\ kHz$

$R = 40\ \Omega$

$\varepsilon = 10$

$\varepsilon_0 = 8.85\ \cdot 10^{-12}\ As\ V^{-1} m^{-1}$

$S = 1\ cm^2$

$$R = \sigma \frac{l}{S}$$

$$C = \varepsilon\varepsilon_0 \frac{S}{l}$$

$$\tau = \frac{1}{2\pi\ \nu_{peak}} = RC = \varepsilon\varepsilon_0\sigma$$

therefore, $\sigma = \frac{1}{2\pi\ \nu_{peak}\ \varepsilon\ \varepsilon_0}$

and $l = \frac{RS}{\sigma} = RS\ 2\pi\ \nu_{peak}\ \varepsilon\ \varepsilon_0$

References

(1) Stejskal, E. O.; Tanner, J. E. Spin Diffusion Measurements: Spin Echoes in the Presence of a Time-Dependent Field Gradient. *J. Chem. Phys.* **1965**, *42* (1). https://doi.org/10.1063/1.1695690.

(2) Lim, K.; Popovic, J.; Maier, J. Ion Transport and Growth Behavior of Solid Electrolyte Interphases on Li and Na with Liquid Electrolytes Based on Impedance Analysis. *J. Mater. Chem. A* **2023**, *11* (11). https://doi.org/10.1039/d2ta09189e.

(3) Lim, K.; Fenk, B.; Popovic, J.; Maier, J. Porosity of Solid Electrolyte Interphases on Alkali Metal Electrodes with Liquid Electrolytes. *ACS Appl. Mater. Interfaces* **2021**, *13* (43). https://doi.org/10.1021/acsami.1c15607.

(4) Talian, S. D.; Brutti, S.; Navarra, M. A.; Moškon, J.; Gaberscek, M. Impedance Spectroscopy Applied to Lithium Battery Materials: Good Practices in Measurements and Analyses. *Energy Storage Mater.* **2024**, *69* (April). https://doi.org/10.1016/j.ensm.2024.103413.

(5) Drvarič Talian, S.; Moškon, J.; Dominko, R.; Gaberšček, M. The Pitfalls and Opportunities of Impedance Spectroscopy of Lithium Sulfur Batteries. *Adv. Mater. Interfaces* **2022**, *9* (8). https://doi.org/10.1002/admi.202101116.

(6) Schechter, A.; Aurbach, D.; Cohen, H. X-Ray Photoelectron Spectroscopy Study of Surface Films Formed on Li Electrodes Freshly Prepared in Alkyl Carbonate Solutions. *Langmuir* **1999**, *15* (9). https://doi.org/10.1021/la981048h.

(7) Aurbach, D.; Pollak, E.; Elazari, R.; Salitra, G.; Kelley, C. S.; Affinito, J. On the Surface Chemical Aspects of Very High Energy Density, Rechargeable Li–Sulfur Batteries. *J. Electrochem. Soc.* **2009**, *156* (8). https://doi.org/10.1149/1.3148721.

(8) Hu, Y.; Kong, W.; Li, H.; Huang, X.; Chen, L. Experimental and Theoretical Studies on Reduction Mechanism of Vinyl Ethylene Carbonate on Graphite Anode for Lithium Ion Batteries. *Electrochem. commun.* **2004**, *6* (2). https://doi.org/10.1016/j.elecom.2003.10.024.

(9) Ota, H.; Sakata, Y.; Otake, Y.; Shima, K.; Ue, M.; Yamaki, J. Structural and Functional Analysis of Surface Film on Li Anode in Vinylene Carbonate-Containing Electrolyte. *J. Electrochem. Soc.* **2004**, *151* (11). https://doi.org/10.1149/1.1798411.

(10) Fiedler, C.; Luerssen, B.; Rohnke, M.; Sann, J.; Janek, J. XPS and SIMS Analysis of Solid Electrolyte Interphases on Lithium Formed by Ether-Based Electrolytes. *J. Electrochem. Soc.* **2017**, *164* (14). https://doi.org/10.1149/2.0851714jes.

(11) Xiong, S.; Xie, K.; Diao, Y.; Hong, X. Characterization of the Solid Electrolyte Interphase on Lithium Anode for Preventing the Shuttle Mechanism in Lithium-Sulfur Batteries. *J. Power Sources* **2014**, *246*. https://doi.org/10.1016/j.jpowsour.2013.08.041.

(12) Peled, E. The Electrochemical Behavior of Alkali and Alkaline Earth Metals in Nonaqueous Battery Systems—The Solid Electrolyte Interphase Model. *J. Electrochem. Soc.* **1979**, *126* (12). https://doi.org/10.1149/1.2128859.

(13) Dedryvère, R.; Leroy, S.; Martinez, H.; Blanchard, F.; Lemordant, D.; Gonbeau, D. XPS Valence Characterization of Lithium Salts as a Tool to Study Electrode/Electrolyte Interfaces of Li-Ion Batteries. *J. Phys. Chem. B* **2006**, *110* (26). https://doi.org/10.1021/jp061624f.
